# Recent advances of cancer chemodynamic therapy based on Fenton/Fenton-like chemistry

**DOI:** 10.1039/d1sc05482a

**Published:** 2021-11-29

**Authors:** Changyu Cao, Xiaorui Wang, Nan Yang, Xuejiao Song, Xiaochen Dong

**Affiliations:** Key Laboratory of Flexible Electronics (KLOFE), Institute of Advanced Materials (IAM), School of Physical and Mathematical Sciences, Nanjing Tech University (NanjingTech) Nanjing 211800 China xjsong@njtech.edu.cn iamxcdong@njtech.edu.cn

## Abstract

Applying Fenton chemistry in the tumor microenvironment (TME) for cancer therapy is the most significant feature of chemodynamic therapy (CDT). Owing to the mild acid and overexpressed H_2_O_2_ in TME, more cytotoxic hydroxyl radicals (˙OH) are generated in tumor cells *via* Fenton and Fenton-like reactions. Without external stimulus and drug resistance generation, reactive oxygen species (ROS)-mediated CDT exhibits a specific and desirable anticancer effect and has been seen as a promising strategy for cancer therapy. However, optimizing the treatment efficiency of CDT in TME is still challenging because of the limited catalytic efficiency of CDT agents and the strong cancer antioxidant capacity in TME. Hence, scientists are trying their best to design and fabricate many more CDT agents with excellent catalytic activity and remodeling TME for optimal CDT. In this perspective, the latest progress of CDT is discussed, with some representative examples presented. Consequently, promising strategies for further optimizing the efficiency of CDT guided by Fenton chemistry are provided. Most importantly, several feasible ways of developing CDT in the future are offered for reference.

## Abstract 2

Recent advances in cancer chemodynamic therapy based on Fenton chemistry are reviewed, including the working mechanism, characteristics, and strategies for optimizing treatment efficiency.

## Graphical abstract

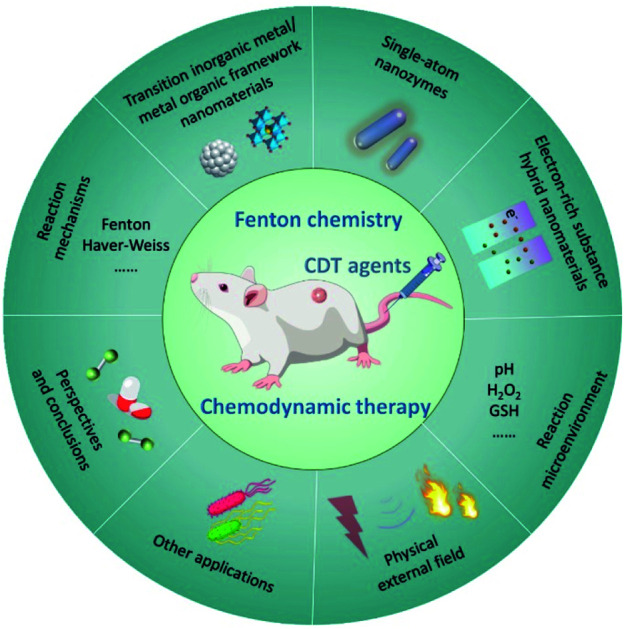

## 1. Introduction

Cancer is one of the most hazardous diseases in the world.^1,2^ The integration of nanotechnology with modern biology and medicine has provided numerous opportunities for tumor therapy with great clinical significance in this century. As cancer cells are more sensitive to the levels of reactive oxygen species (ROS),^3,4^ many nanomaterials using ROS-mediated cancer treatment mechanisms have emerged,^5–16^ for instance, applying nanomaterials for photodynamic therapy (PDT), sonodynamic therapy (SDT), traditional chemotherapeutic drug delivery, enhanced radiotherapy (RT), and so on. Furthermore, the tumor microenvironment (TME) has been considered as an important factor that significantly affects the above-mentioned treatment effects and more and more studies have begun to incorporate the tumor microenvironment into the diagnosis and treatment of tumors.^17^ In brief, the features of TME mainly include mild acidity, hypoxia, overexpressed H_2_O_2_ and glutathione (GSH, major antioxidants in TME), abnormal vessels, high nutrient consumption, *etc.* Therefore, ROS-mediated tumor therapy with specific TME-responsive will become the trend of cancer therapy.

By specifically combing TME and the Fenton/Fenton-like reactions, chemodynamic therapy (CDT), which utilizes chemodynamic therapeutic agents to convert internal hydrogen peroxide (H_2_O_2_) into toxic hydroxyl radicals (˙OH) for cancer cell killing, stands out from many other proof-in-concept ROS-related cancer therapy strategies, such as traditional chemotherapy, radiotherapy, PDT, and SDT.^18–22^ This could be attributed to its (1) higher specific response to H_2_O_2_, (2) no external field penetration depth restriction, (3) fewer side effects on normal tissues, (4) more desirable ROS generation ability, (5) no drug-resistance, device limitations, and external stimulation. Thus, CDT exhibits a promising future for clinical transformation and other practical applications. Throughout CDT, Fenton/Fenton-like reactions play a critical role in determining the treatment efficiency. Generally, the performance of CDT in TME could be optimized by reducing the reaction potential of Fenton/Fenton-like reactions. From this angle, two strategies should be carefully considered to optimize the performance of CDT in TME. On one hand, chemodynamic agents (Fenton agents) with more active sites should be rationally designed and constructed for catalyzing H_2_O_2_ into more ˙OH. On the other hand, the TME (pH, low H_2_O_2_ content, and over-expressed reduced substance) could be remodeled to provide more suitable reaction conditions for Fenton/Fenton-like reactions.

**Fig. 1:**
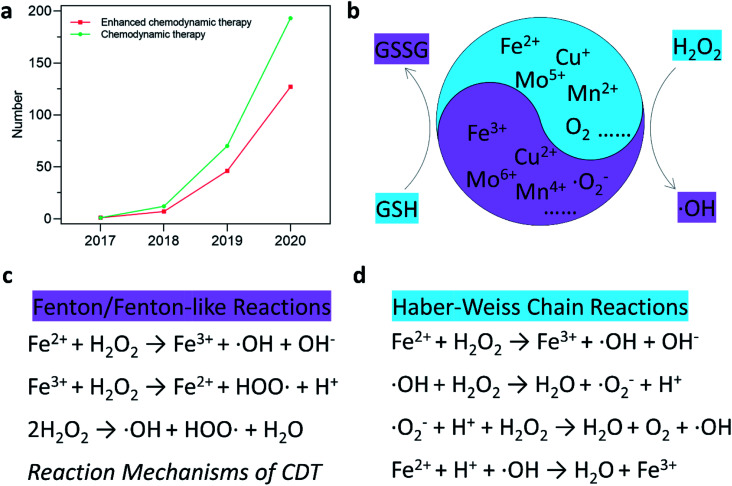
(a) Number of published research articles for “chemodynamic therapy” and “enhanced chemodynamic therapy” from Web of Science. (b) Schematic illustration of chemodynamic therapy mechanism. (c and d) Specific reaction mechanism of Fenton/Fenton-like reactions and Haber–Weiss Chain reactions.

In this perspective, the foundational therapeutic mechanism of chemodynamic therapeutic agents in TME based on Fenton chemistry is supplied in detail. Subsequently, the current progress of CDT is discussed with some representative examples. Based on the hinders that CDT faces till now, corresponding solutions are proposed – the selection of suitable CDT agents, the regulation of reaction conditions (increased proton/H_2_O_2_ level, decreased GSH concentration, reduced pH value, *etc.*), and the help of external stimulation (photo, ultrasound, magnetic, *etc.*). The comparisons of different kinds of CDT agents used in different synergetic treatment methods are also provided. The application scenario of CDT is still limited to tumor treatment, other biological fields could be considered, like antibacterial therapy. Notably, a deeper understanding of *in vivo* therapeutic mechanisms and biosafety should be carefully studied. Last but not least, the importance of the introduction of imaging technologies and external stimulation to support or enhance CDT is seriously underestimated, this would also be discussed in the perspective. We conceive that CDT can be a promising candidate for cancer clinical treatment owing to its rapid development (Fig. 1a).

## 2. The therapeutic mechanism of CDT agents

Current therapeutic mechanisms of CDT agents are systematically offered in this section. Since the Fenton/Fenton-like reactions were first invented by Henry J. Fenton in the 1890s, the reactions have flourished in the field of contaminants or water treatment. In addition, various chemodynamic therapeutic agents, such as Fe, Cu, Mn, Mo, Cr, Ti, Co, Al, Ce, and Ru-based nanomaterials,^23–33^ have been well developed and applied in cancer CDT (Fig. 1b). Essentially, CDT typically relies on the reaction between Fe^2+^ and H_2_O_2_

1 Fe^2+^ + H_2_O_2_ → Fe^3+^ + ˙OH + OH^−^,

(Fig. 1c), Haber–Weiss reaction (˙O_2_^−^ + H_2_O_2_ → ˙OH + OH^−^ + O_2_) and Haber–Weiss chain reaction (˙O_2_^−^ + H_2_O_2_ + H^+^ → ˙OH + H_2_O + O_2_) (Fig. 1d) to generate highly oxidative ˙OH, thus leading to DNA damage, lipid peroxidation, and other damage of biomacromolecules.^34^ Generally speaking, these reactions are relatively complicated and maintain a series of reactions, such as the initiation, propagation, and termination of reaction parts. Specifically, for the reaction mechanism of the Fenton reaction, ˙OH is firstly produced through the reaction between Fe^2+^ and H_2_O_2_ in eqn (1). Subsequently, the produced Fe^3+^ is reduced by antioxidant GSH in TME to reproduce Fe^2+^

2 Fe^3+^ + GSH → Fe^2+^ + GSSG (glutathione disulfide),

causing a replenishment of Fe^2+^. However, some significant factors that can inhibit the reaction efficiency are listed as follows: (1) given the concentration of endogenous Fenton reactant H_2_O_2_, overproduced in TME (<100 μM) because of the abnormal growth and dysfunction in metabolism,^35^ is still not enough for the requirement of optimal CDT. Considering H_2_O_2_ consumption, a large amount of H_2_O_2_ is urgently needed to achieve a desirable CDT performance in clinical application. (2) Although the mild acidic TME is beneficial for the Fenton reaction, there is a narrow optimum pH range for the reaction, for instance, the optimal pH range of the Fe Fenton reaction is 3–4.^36^ In detail, when the pH is above 3, Fe(OH)_2_ is beginning to generate from Fe^2+^. Furthermore, the amount of Fe(OH)_2_ reaches the maximum when the pH value is 4, at which point the reaction activity of Fe(OH)_2_ is much higher than that of Fe^2+^, thus largely hampering further Fenton reactions. Under this circumstance, matching the pH range for optimizing CDT seems necessary and attractive. (3) The reducing substance like GSH in TME is a double-edged sword, which reacts with high valence catalyst metal ion (Fe^3+^) to low valence state (Fe^2+^) with higher Fenton reaction activity (eqn (2)) while prevents cancer cells from oxidative damage. Hence, balancing the level of reducing substance is also significant. (4) For the heterogeneous Fenton/Fenton-like reactions, element Fe is stabilized within the nanomaterial structures and the redox cycle between the different valence states of Fe is a critical role for high efficient Fenton/Fenton-like reactions. Hence, promoting the rate-limiting process in Fenton/Fenton-like reactions is a motivation for designing more efficient Fenton catalysts and reaction procedures. (5) With the rapid development of Fenton/Fenton-like reactions, whether homogenous or heterogenous, peroxymonosulfate and persulfate-triggered Fenton/Fenton-like reactions are also proposed to generate ˙OH. Therefore, other substrate-triggered Fenton reactions are also developed recently and become a novel mechanism of Fenton/Fenton-like reactions. In a word, starting from the mechanism, researchers could be inspired to design more powerful agents for CDT in the complicated TME both *in vitro* and *in vivo*. Overall, CDT is in its infancy, and in-depth researches are needed from its special mechanisms.

## 3. CDT therapeutic agents selection

**Fig. 2:**
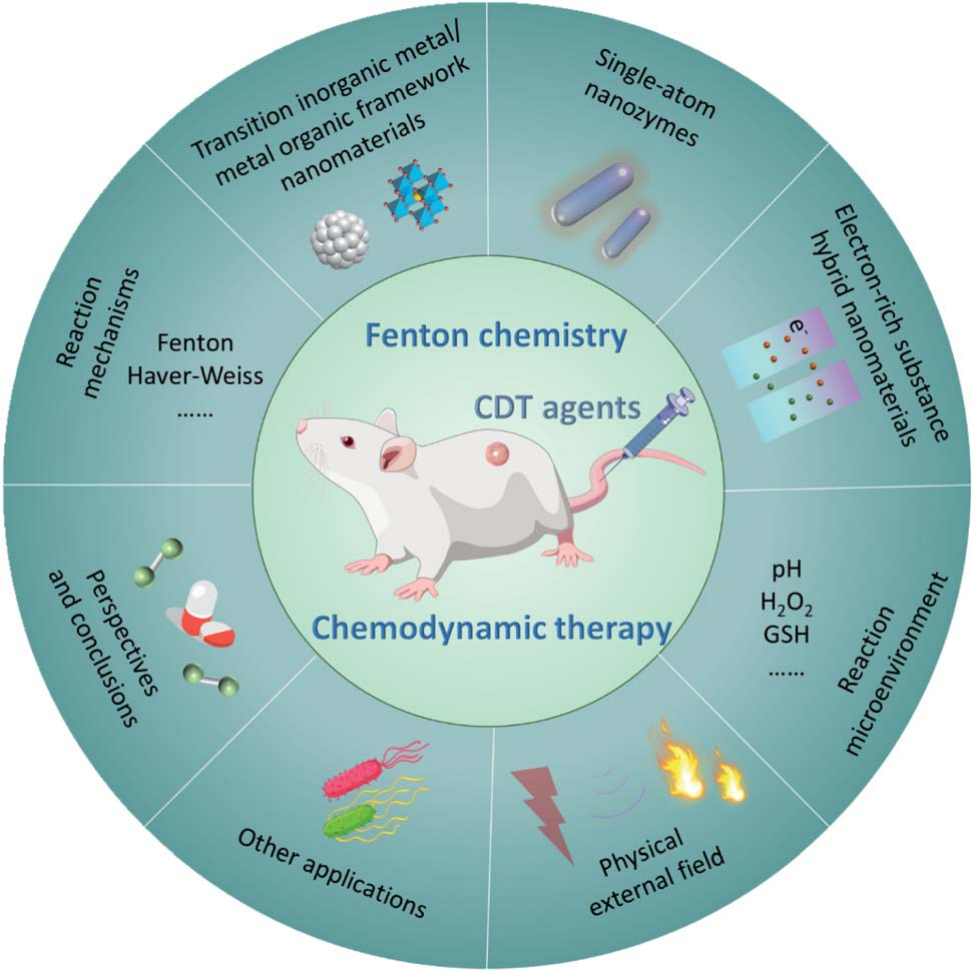
Overall classification and overview of nanomaterials-based chemodynamic therapeutic agents and improved strategies for CDT.

Based on the understanding of the CDT mechanism associated with Fenton chemistry and the major factors that inhibit the catalytic efficiency of Fenton/Fenton-like reactions, researchers are beginning to pay great attention to developing novel CDT therapeutic agents in optimizing the effect of CDT. In this section, various kinds of nanomaterials-based nanoplatforms used for CDT are provided for discussion, including transition metal-based inorganic nanomaterials, organic framework nanomaterials, single-atom Fenton nanozymes, and electron-rich substance hybrid nanomaterials. The design concepts, structures, advantages, and disadvantages of the mentioned CDT agents are summarized for discussion using some representative examples, aiming to provide references for preparing more efficient chemodynamic agents. The classifications of current chemodynamic agents and improved strategies for CDT are summarized in Fig. 2.

### 3.1 Transition metal-based inorganic nanomaterials

The transition metal can de be defined as that an element whose atom has a partially filled d sub-shell, and elemental iron is seen as an important and special element that possesses many unique features. In living organisms, elemental iron is an inherent nutrient element and play a crucial role in a series of biological process, for instance, amino acid metabolism, polyunsaturated fatty acid, and enzyme biosynthesis. In addition, the previous study has evidenced that iron ions can induce cell death *via* ferroptosis without causing chemotherapeutic multidrug resistance, thus being explored as a promising candidate for cancer therapy.^37–40^ Fenton catalyst fabricated by superparamagnetic iron oxide was used for improving the treatment efficiency of anticancer drugs named β-lapachone by increasing the generation of ROS in 2013.^41^ Food and Drug Administration (FDA) also approved ferumoxytol and other iron oxide nanoparticles for cancer treatment because of Fenton reaction-induced ROS generation.^42^ Taken together, applying Fe-based nanomaterials in cancer therapy has experienced a long history and achieved great success. Recognizing that Fe Fenton reaction is a conventional homogeneous Fenton/Fenton-like reaction process, along with the free iron ions converting H_2_O_2_ to ˙OH. Based on this, the way in increasing the CDT efficiency is to improve the number of free iron ions in TME. Besides, the parameters including decreasing pH, increasing localized temperature and H_2_O_2_ dosage, as well as consuming GSH of the whole Fenton/Fenton-like reactions should be also considered to achieve enhanced therapeutic efficiency.

**Fig. 3:**
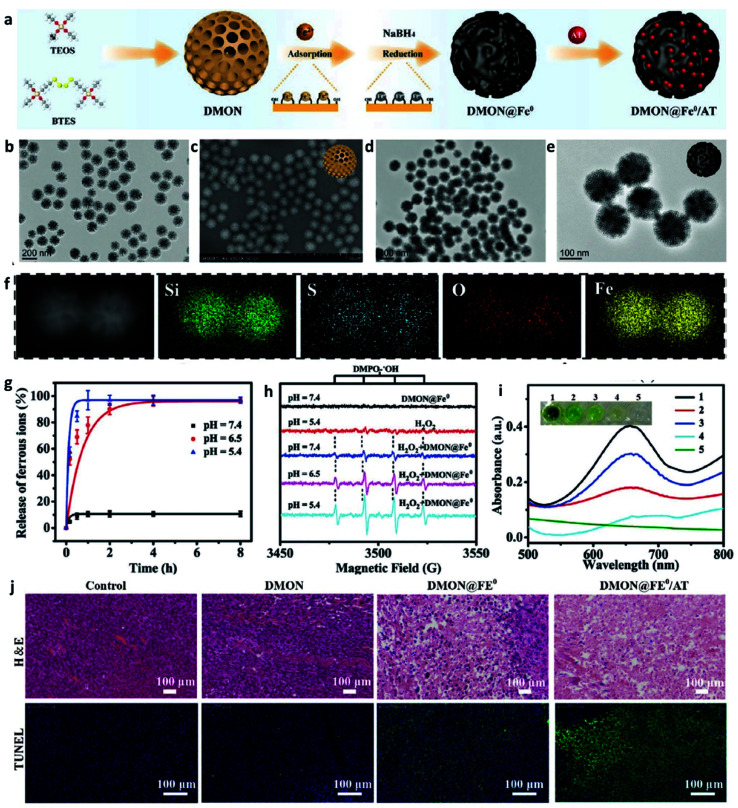
(a) Schematic illustration of the synthesis of DMON@Fe^0^/AT NP (DMON: dendritic mesoporous organic silica nanoparticles, AT: catalase inhibitor (3-amino-1,2,4-triazole)). (b and c) TEM and SEM images of DMON. (d and e) TEM images of DMON@Fe^0^/AT NP. (f) Elemental mappings of DMON@Fe^0^/AT NP. (g) Release curve of ferrous ions in different pH (pH = 5.4, 6.5, and 7.4). (h) EPR spectra of DMON@Fe^0^/AT NP under different conditions. (i) Absorbance changes of TMB probe by DMON@Fe^0^/AT in different pH ((1) pH 5.4, (2) 6.5, (3) 7.4, (4) H_2_O_2_ control, (5) PBS control). (j) H&E and TUNEL staining images of tumor slices treated with different groups. Reproduced with permission from ref. 43. Copyright 2021, Wiley-VCH.

Given the intrinsic weakly acidity of the TME, pH-responsive/sensitive Fe-containing nanomaterials have been developed, which could specifically release Fe^2+^/Fe^3+^ couples in TME to trigger the Fenton/Fenton-like reaction to kill cancer cells. For instance, in 2021, Bu's group successfully co-loaded amorphous elemental Fe^0^ and catalase inhibitor (2-amino-1,2,4-triazole, AT) into a novel biodegradable nanocarrier DMON (S–S bond-rich dendritic mesoporous organic silica nanoparticle) to synthesize DMON@Fe^0^/AT NP (Fig. 3a–f), which was rapidly ionized in the acidic TME condition to release Fe^2+^ ions for the specific killing of cancer cells.^43^ As evidenced in Fig. 3g, the release rate of ferrous ions at a pH of 5.4 was much higher than that observed when the pH was at 7.4. The EPR spectra in Fig. 3h and absorbance change of TMB in Fig. 3i also confirmed the above-mentioned result. Consequently, owing to the capacity of DMON@Fe^0^/AT NP for selective ferrous ions release, thus ensuring an enhanced CDT efficiency *in vivo* (Fig. 3j). In addition, many other pH-sensitive iron-based nanomaterials, such as iron oxides, FePt, [FeO(OH)_*n*_], *etc.*, have also been prepared as chemodynamic therapeutic agents by increasing the release of catalytic Fe^2+^ ions in TME.

**Fig. 4:**
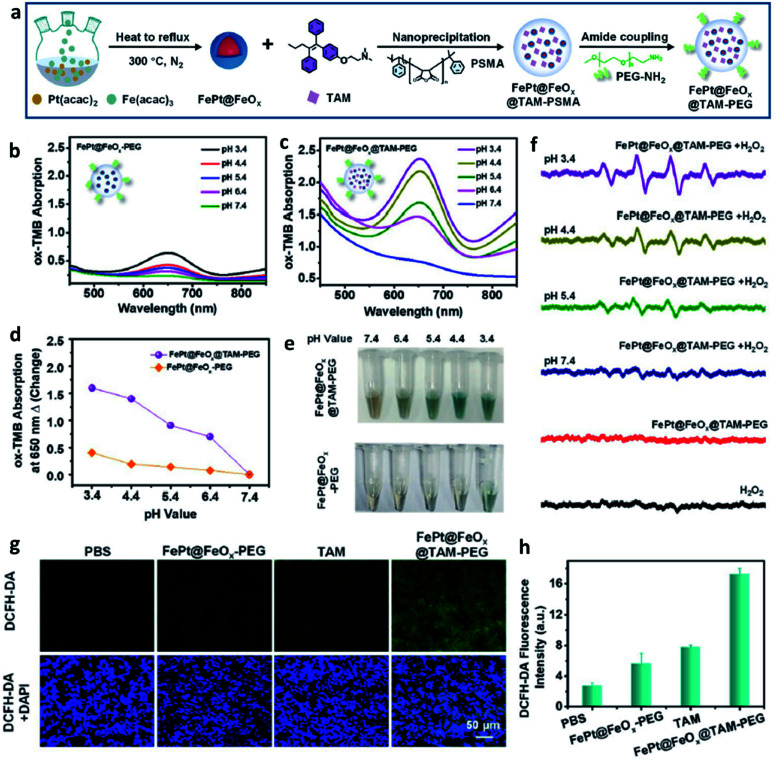
(a) Schematic synthetic process of FePt@FeO_*x*_@TAM-PEG NP. (b–e) Measurement of ˙OH generation *via* TMB probe for FePt@FeO_*x*_-PEG or FePt@FeO_*x*_@TAM-PEG NP in different pH (pH = 3.4, 4.4, 5.4, 6.4, and 7.4). (f) ESR spectra of different groups at different pH. (g) ROS staining images using DCFH-DA probe of tumor slices for different groups (green indicates ROS and blue indicates cell nucleus). (h) The corresponding quantification of fluorescent intensity from (g). Reproduced with permission from ref. 44. Copyright 2021, Wiley-VCH.

Furthermore, based on the understanding of heterogeneous Fenton/Fenton-like reactions, which typically use iron ions in solid-phase materials to catalyze the conversion of H_2_O_2_ into ˙OH, some heterogeneous nanocatalysts are beginning to be applied in CDT. This could be attributed to the reason that these nanomaterials can work over a wide range of pH instead of being limited by the pH condition of TME. For instance, in 2021, Song's group provided FePt@FeO_*x*_@TAM-PEG (TAM: tamoxifen) nanocatalysts to serve as novel exploited outstanding heterogeneous catalysts (Fig. 4a), which could be used for H^+^ self-accumulated cancer chemodynamic therapy derived by heterogeneous Fenton/Fenton-like reaction with boosting catalytic efficiency.^44^ As shown in Fig. 4b–f, FePt@FeO_*x*_@TAM-PEG NP exhibited a better ROS generation ability under the same pH condition compared with FePt@FeO_*x*_-PEG because of the self-supplied H^+^ by TAM, thus achieving a better chemodynamic therapeutic performance in cell assays (Fig. 4g). Such Fe-based smart nanoplatform that can self-generate H^+^ significantly boost the ability to generate ROS, so the weakly acidic conditions in TME would no longer be a limiting factor of the efficiency of CDT.

**Fig. 5:**
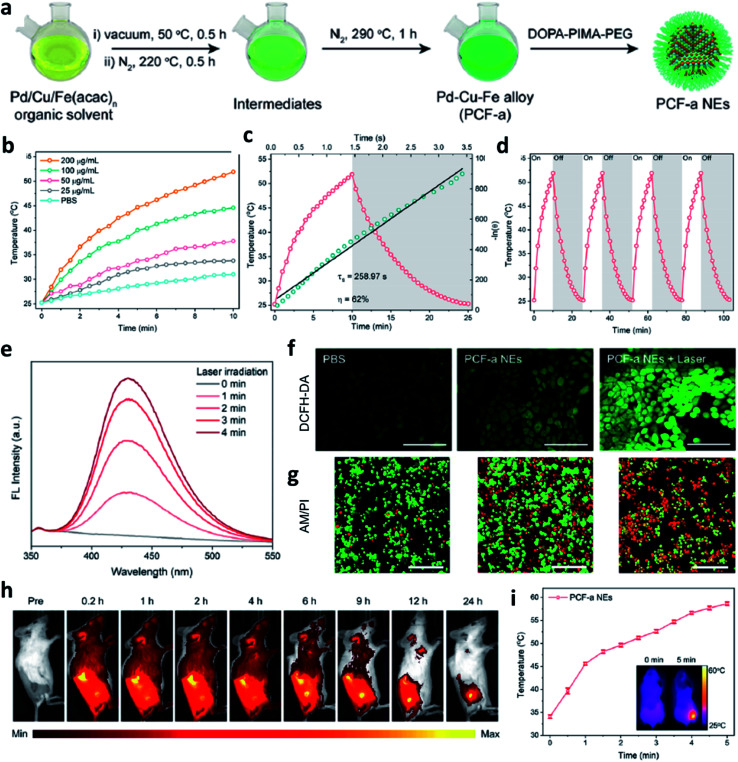
(a) Schematic synthetic process of PCF-a NEs (P: Pd, C: Cu, F: Fe, a: alloy, NE: nanozyme). (b–d) Photothermal conversion process, heating and cooling curves, and photothermal stability of PCF-a NEs. (e) Photothermal-enhanced ˙OH generation of PCF-a NEs. (f) CLSM images of 4T1 cells stained by DCFH-DA probe to detect ROS generation. Scar bar: 100 μm. (g) Live-dead staining images of 4T1 cells using AM/PI dyes (green indicates live cells and red indicates dead cells). Scar bar: 100 μm. (h) Representative fluorescence images of 4T1 tumor-bearing mice after intravenous injection of Cy5.5-labeled PCF-a NEs. (i) Temperature change of tumor site after irradiated with 808 nm laser. Inset: the infrared thermal images of mice. Reproduced with permission from ref. 45. Copyright 2021, American Chemical Society.

Apart from the factor of pH value, as we all know, a temperature rise could lead to an increase in the kinetic energy of molecules and the emergence of photothermal agents could effectively convert photo energy into localized heat. Therefore, combing photothermal agents with Fe-based nanomaterials can also achieve the accelerated release of Fe^2+^ due to the thermal effect to obtain an enhanced CDT efficiency. Besides, photothermal agents-induced thermal energy can also normalize the tumor vasculature to further alleviate hypoxia in TME, finally supplying oxygen for continuous H_2_O_2_ generation by superoxide dismutase. Hence, Fe-based nanomaterials together with photothermal agents have also been seen as a promising method for enhancing the performance of CDT. It is worthy to note that the local production of heat generated by light energy like laser is restricted due to the insufficient penetration and deposition in human tissues. For example, in 2021, Zhao's group designed and prepared an ultrasmall trimetallic (Pd, Cu, and Fe) alloy nanozyme (nanomaterial with enzyme-like activities) named PCF-a NEs containing dynamic active-site synergism, thus showing a cascade peroxidase mimicking activities (Fig. 5a).^45^ Notably, PCF-a NEs exhibited a desirable photothermal performance with high photothermal conversion efficiency (62%) (Fig. 5b–d). Owing to the excellent photothermal effect, PCF-a NEs showed a photothermally augmented CDT efficiency (Fig. 5e), which is greatly beneficial to generate ˙OH (Fig. 5f) for effectively killing cancer cells (Fig. 5g). As shown in Fig. 5h and i, after PCF-a NEs were accumulated in the tumor region and then irradiated by 808 nm laser, the localized temperature rose from 34 to 58 °C, thus realizing a photothermal-boosting chemodynamic therapy.

**Fig. 6:**
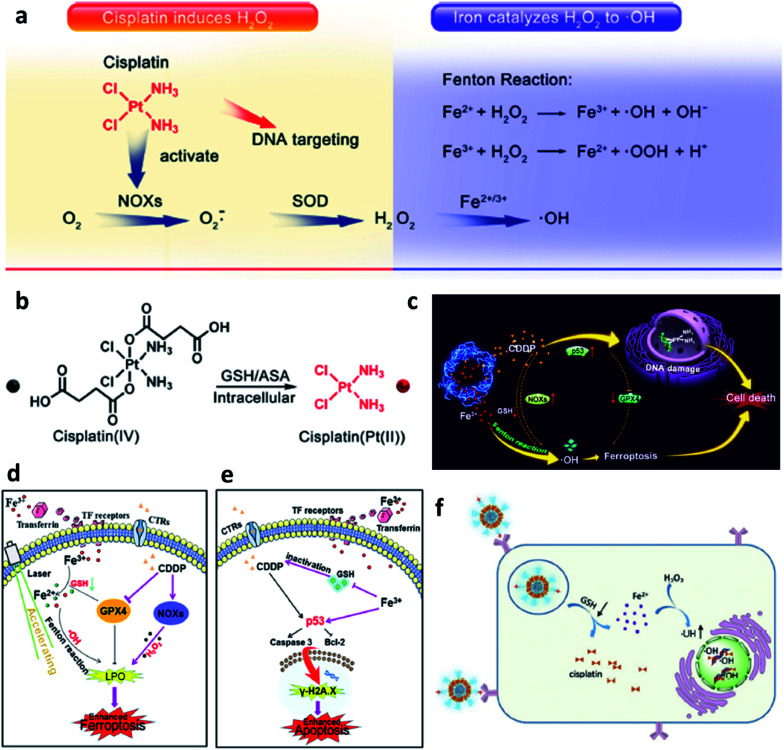
(a) Cisplatin(Pt(ii)) activates nicotinamide adenine dinucleotide phosphate (NADPH) oxidase (NOX), which can catalyze the formation of superoxide (O_2_˙^−^) and H_2_O_2_ from oxygen, iron ions catalyze the Fenton reaction to converse H_2_O_2_ into cytotoxic ˙OH. (b) Cisplatin(iv) can be turned into cisplatin(Pt(ii)) by reacting with intracellular GSH. Reproduced with permission from ref. 46. Copyright 2017, American Chemical Society. (c) Schematic illustration of synergistic therapy by combing cisplatin and Fe-based nanomaterials along with consuming the GSH. (d) Schematic illustration of cisplatin-enhanced CDT *via* adjusting relevant proteins. (e) Schematic illustration of CDT in CDDP-induced anticancer therapy. Reproduced with permission from ref. 47. Copyright 2020, American Chemical Society. (f) Schematic illustration of GSH consumption induced synergistic cisplatin release and ˙OH generation and ˙OH enhanced DNA damage. Reproduced with permission from ref. 48. Copyright 2020, Wiley-VCH.

Last but not least, to make up for the shortage of H_2_O_2_ dosage and increase the consumption of GSH in TME, Lin's group,^46^ Yu's group,^47^ and Liu's group^48^ all combined chemotherapeutic prodrug cisplatin(iv) with Fe-based nanomaterials for enhancing CDT efficiency. As shown in Fig. 6, on one hand, cisplatin(iv) with the enzyme-like activity could effectively be reduced by GSH to produce cisplatin(Pt(ii)) to make damage to DNA. The depletion of GSH would further enhance the ROS-mediated CDT because of the destroy of antioxidant system. On another hand, produced cisplatin(Pt(ii)) can effectively trigger the activation of nicotinamide adenine dinucleotide phosphate (NADPH) oxidase (NOX), which can then convert oxygen into superoxide radical (O_2_˙^−^) and its downstream H_2_O_2_. The replenishment of H_2_O_2_ makes the CDT more efficient and sustainable. The combination of chemotherapeutic prodrug and Fe-based catalysts provides a desirable selection for CDT agents.

**Fig. 7:**
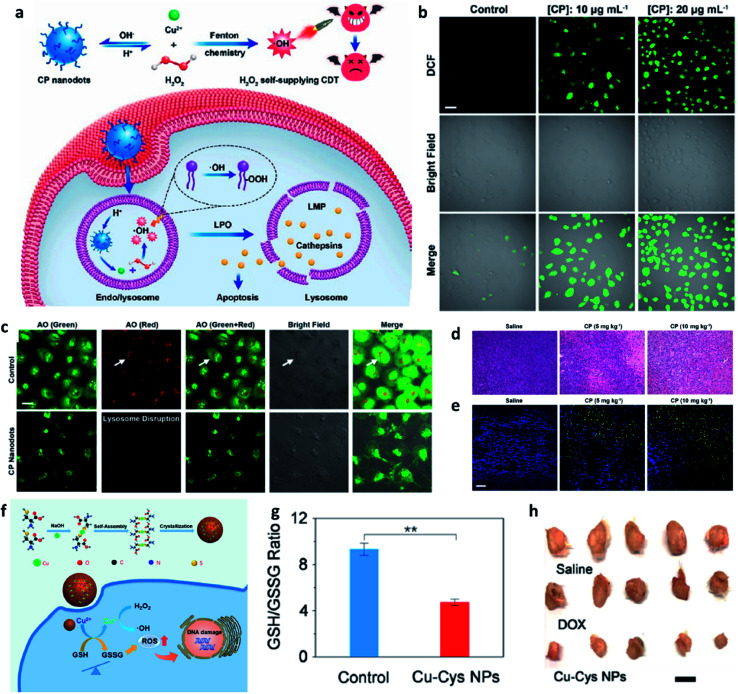
(a) Schematic synthetic process of copper peroxide (CP) nanodots for H_2_O_2_ self-supplying enhanced CDT. (b) Fluorescence images of DCFH-DA stained cancer cells (U87MG cell line). Scar bar: 50 μm. (c) CLSM images of acridine orange (AO)-stained cancer cells (U87MG) treated with or without CP nanodots. Scar bar: 20 μm. (d) H&E staining images and (e) TUNEL staining images of tumor slices treated with different groups. Scar bar: 50 μm. Reproduced with permission from ref. 49. Copyright 2019, American Chemical Society. (f) Schematic synthetic process of copper-cysteine mercaptide nanoparticles (Cu-Cys NPs) for copper-involving nanoformulation mediated CDT. (g) GSH/GSSG ratio in cancer cells (MCF-7R) incubated with or without Cu-Cys NPs. (h) Representative photographs of tumors from MCF-7R tumor-bearing mice treated with different groups. Scar bar: 50 μm. Reproduced with permission from ref. 50. Copyright 2019, American Chemical Society.

Apart from the excellent catalytic effect of ferrous ions, some other transition metal ions (*i.e.*, Cu, Mn, Mo, Co, W, *etc.*) can also serve as catalytic ions to participate in peroxide-initiated ˙OH generation to culminate in ROS-mediated CDT. Similar to the Fe Fenton process, the types of other metal-based treatment processes can be referred to as the typical Fenton/Fenton-like process. Until now, many different kinds of transition metal-based nanocatalysts have been developed as chemodynamic therapeutic agents for enhanced CDT *via* typical homogeneous or heterogeneous Fenton/Fenton-like reactions. For example, in 2019, Chen's group firstly reported the preparation of copper peroxide nanodot, which was used as an activable chemodynamic agent for enhancing CDT efficiency by self-supplying H_2_O_2_ (Fig. 7a–e).^49^ After endocytosis into cancer cells, acidic TME of endo/lysosomes would promote the dissociation of copper peroxide nanodots, thus allowing the continuous release of copper ions and H_2_O_2_ along with a Fenton reaction between them. The produced ˙OH would then induce lysosomal membrane permeabilization *via* lipid peroxidation and finally cause tumor cell death through a lysosome-related pathway. Chen's work provides a typical example for fabricating Cu-based Fenton catalysts to improve CDT efficacy. Furthermore, from the angle that the combination of GSH consumption with Fenton nanocatalysts can effectively improve the efficiency of CDT, Bu's group demonstrated copper-amino acid mercaptide nanoparticle (Cu-Cys NP) *via* the self-assembled method for GSH-activated and H_2_O_2_-reinforced enhanced CDT (Fig. 7f).^50^ After endocytosis into cancer cells, the released Cu-Cys NP could rapidly react with local GSH for inducing GSH depletion(Fig. 7g), while the Cu^2+^ was reduced to Cu^+^ for reacting with endogenous H_2_O_2_ to generate ˙OH, thus killing cancer cells. The *in vivo* result confirmed that Cu-Cys NP can efficiently inhibit the development of tumors. This is also a typical sample for demonstrating Cu-based nanocatalysts for CDT. Therefore, the combination of other transition metal ions with TME is also a promising approach for progressing CDT efficiency.

### 3.2 Organic framework nanomaterials

In addition to transition metal inorganic nanomaterials, nanomaterials with metal–organic frameworks (MOFs), covalent-organic frameworks (COFs), and macromolecular nanoparticles (an assembly of small molecules without periodic network structures) also have great potential to be applied for chemodynamic therapy. Typically, organic framework nanomaterials are porous materials that possess high surface areas. Compared with inorganic nanomaterials, organic framework materials exhibit multifunctionalities because of the combination of metal ions and organic functional entities, thus achieving many different goals in one nanoplatform. In addition, due to their competitive flexibility, biocompatibility, and better responsiveness, many organic framework nanomaterials have been designed and prepared for CDT and imaging.

#### 3.2.1 Metal–organic frameworks (MOFs) nanomaterials

**Fig. 8:**
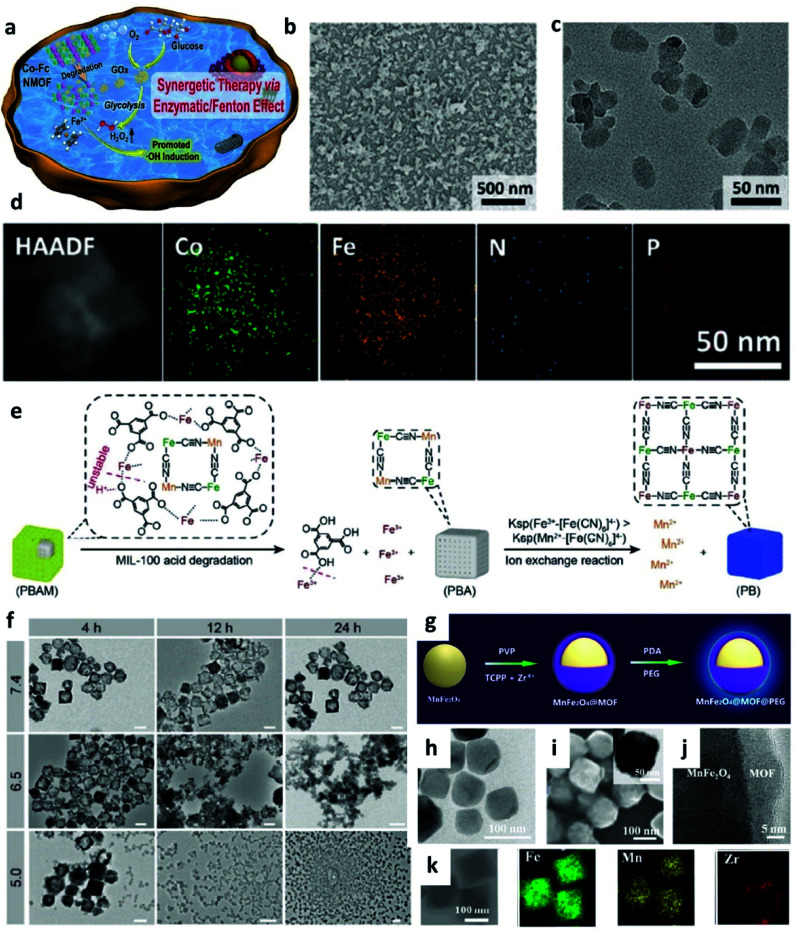
(a) Schematic synthetic process of Co-Fc@GOx (nanoscale Co-ferrocene metal–organic framework, Co-Fc NMOF) as a cascade Fenton and enzymatic reaction nanoplatform for promoted ˙OH generation. (b–d) SEM, TEM image, and elemental mapping image of Co-Fc@GOx NMOF. Reproduced with permission from ref. 51. Copyright 2020, Wiley-VCH. (e) Schematic illustration of acid degradation and the rapid ion exchange reaction during the nanotheranostic agents (NTAs) synthesis process. *K*_sp_: stability constant. (f) TEM images of PBAM (a MIL-100 (Fe)-coated Prussian blue (PB) analogue (K_2_Mn[Fe(CN)_6_])) in different pH conditions (pH = 5.0, 6.5, and 7.4). Scar bar: 100 nm. Reproduced with permission from ref. 52. Copyright 2020, Wiley-VCH. (g) Schematic synthetic process of MnFe_2_O_4_@MOF. (h) TEM image of MnFe_2_O_4_ NP. (i) SEM, TEM (inset), (j) high-resolution TEM, and (k) elemental mapping images of MnFe_2_O_4_@MOF. Reproduced with permission from ref. 53. Copyright 2019, Wiley-VCH.

Metal–organic frameworks (MOFs) are star materials because of their attractive features and a class of compounds consisting of metal ions coordinated to organic ligands to multiple (one-, two-, or three-) dimensional structures. In addition, MOFs often own multiple functions with enzyme-like activity and has been recognized that MOFs are suitable candidates for chemodynamic therapy. For instance, in 2020, Han's group synthesized a nanoscale Co-ferrocene metal–organic framework (Co-Fc NMOF) which was then combined with the neutral enzyme glucose oxidase (GOx) to co-construct a cascade enzymatic/Fenton nanoplatform for enhanced CDT (Fig. 8a–d).^51^ Considering the acid at TME and limited intracellular concentration of H_2_O_2_, Co-Fc NMOF could not only serve as a versatile and high efficient carrier of GOx to re-modulate the content of H_2_O_2_*via* the enzyme–catalytic reactions but also exhibited a desirable Fenton effect for the production of highly toxic ˙OH. This work proves that MOF could be seen as an excellent delivery cargo to carry GOx in tumor sites, for making up for the shortage of H_2_O_2_ and breaking up the limitation of CDT, which is attributed to its porous structure.

It is well known that nanotheranostic agents that combine diagnostic abilities and therapeutic functions in one nanoplatform have great potential for precision medicine and personalized treatment. For example, in 2020, Zhang's group reported a pro-nanotheranostic agent (precursor of nanotheranostic agent) activation strategy for enhanced CDT with specificity. This pro-nanotheranostic agent was named PBAM, which is constructed by the MIL-100 (Fe)-coated Prussian blue (PB) analogue (K_2_Mn[Fe(CN)_6_]) (Fig. 8e).^52^ When meeting the mildly acidic TME, PBAM can be activated to form PB nanoparticles (serve as photothermal agents) accompanied by the release of Mn^2+^ ions because of the internal fast ion exchange, thus resulting in the “on” state for specifical CDT (Fig. 8f). These MOF-based nanotheranostic agents use both the TME acidity and photothermal effect to achieve highly selective treatment with excellent specificity.

In addition, the strategy of depleting GSH is also successfully applied in MOF-based nanocatalysts, for instance, in 2019, Zhang's group reported a biocompatible nanoplatform (MnFe_2_O_4_@MOF) with the capacity to continuously and simultaneously regulate TME for enhanced CDT (Fig. 8g–k).^53^ Notably, porphyrin-based MOF here severs as a photosensitizer, and MnFe_2_O_4_ acts as a nanocatalyst. Benefiting from both catalase-like and glutathione peroxidase-mimicking activities, once accumulated in the tumor, MnFe_2_O_4_@MOF could not only effectively produce oxygen for H_2_O_2_ generation, but also consume the overexpressed GSH for decreasing the antioxidant level in TME, thus significantly improving the treatment efficiency.

Additionally, zeolitic imidazolate frameworks (ZIFs) are a class of MOF that are topologically isomorphic with zeolites and composed of tetrahedrally-coordinated transition metal ions (*e.g.* Fe, Cu, Zn, and Co) linked by imidazolate connectors. Owing to their well acid-responsive release behavior and multiple functions, ZIFs are also beginning to be applied in specific CDT. Taken together, MOF-based nanomaterials with porous, multifunctionality and highly catalytic activities also have great potential for CDT.

#### 3.2.2 Covalent-organic frameworks (COFs) nanomaterials

**Fig. 9:**
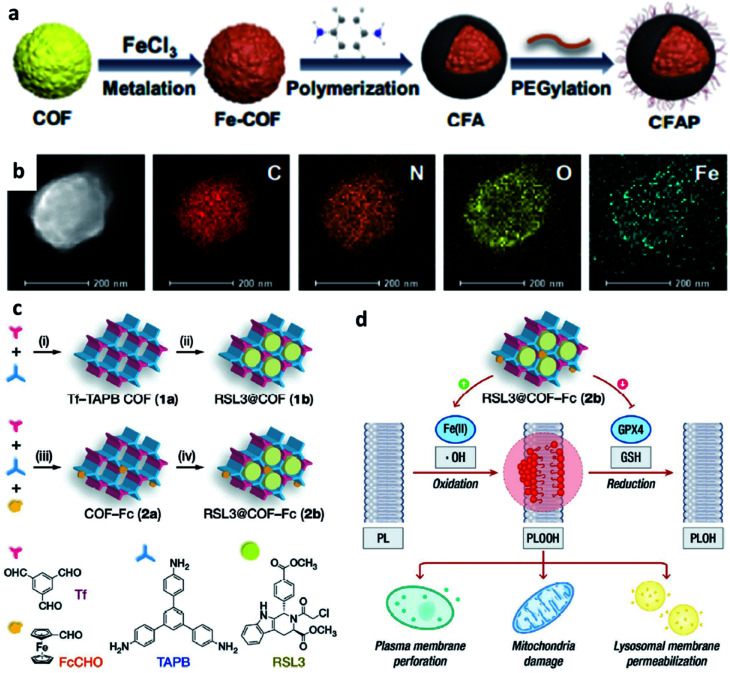
(a) Schematic synthetic process of CFAP (PEGylated CFA, CFA: after being metalized with FeCl_3_, *p*-phenylenediamine is then polymerized on the COF). (b) Elemental mapping of CFAP. Reproduced with permission from ref. 54. Copyright 2020, American Chemical Society. (c) Schematic synthetic process of COF-based nanomaterials (1b: RSL3@COF, 2b: RSL3@COF-Fc) under different reaction conditions ((i) CH_3_COOH, acetonitrile; (ii) RSL3, ethanol; (iii) CH_3_COOH, acetonitrile; and iv, RSL3, ethanol). (RSL3 indicates a small molecule inhibitor of glutathione peroxidase 4 (GPX4) named methyl (1*S*,3*R*)-2-(2-chloroacetyl)-1-(4-(methoxycarbonyl)phenyl)-2,3,4,9-tetrahydro-1*H*-pyrido[3,4-*b*]indole-3-carboxylate). (d) By blocking GPX4-mediated reduction of PLOOH and induce lipid peroxidation to enhance treatment effect. Reproduced with permission from ref. 55. Copyright 2021, Wiley-VCH.

Compared with MOFs, covalent organic frameworks (COF) are made wholely from light elements like H, B, N, C, and O along with extended structures. Due to the characteristics of low density, adjustable and controllable pore size, and large surface area, COFs-based chemodynamic therapeutic agents have also attracted much attention and developed for cancer therapy. For example, in 2020, Pang's group reported a multifunctional COF-based nanocomposite for photothermal-enhanced Fenton reaction to generate more ˙OH (Fig. 9a and b).^54^ In detail, the introduced COF possesses the inherent ability to generate singlet oxygen species under 650 nm laser irradiation and then is combined with FeCl_3_ (Fenton ions resources) and *p*-phenylenediamine (photothermal agents) to achieve specific goals. Herein, the increase of temperature-induced by photothermal effect of *p*-phenylenediamine can effectively accelerate the generation of ˙OH based on the above-mentioned mechanism of Fenton chemistry. Such a COF-based multifunctional nanoplatform offers another selection of nanocatalysts for CDT with adjustable functions.

Similar to MOFs, COFs can also be designed to break the limitation by the highly upregulated and controlled cellular antioxidant defense. For example, in 2021, Dong's group designed and prepared COF-based nanomaterials containing ferrocene (Fc)- and glutathione peroxidase 4 (GPX4) inhibitor-carried nanodrug (RSL3@COF-Fc, Fig. 9c and d).^55^ In brief, commercial RSL3 is a small organic molecule that could inhibit the expression of GPX4 in cells. Upon RSL3@COF-Fc was endocytosed, the released RSL3 would effectively inhibit the expression of GPX4, the core checkpoint of the cancer cell antioxidant system, that could disturb redox homeostasis. While Fc induced ˙OH generation *via* Fenton/Fenton-like reactions, finally resulting in lipid peroxidation. Based on the premise of cell redox dyshomeostasis, effective repair of oxidative damage cannot be achieved even if there is a high content of GSH in the cells, thus allowing toxic ˙OH accumulation. Ultimately, RSL3@COF-Fc led to the plasma membrane, lysosomal, and mitochondrial damage and subsequent ferroptosis of tumor cells, and was less toxic to normal cells. Consequently, this approach significantly results in massive lipid peroxide accumulation, subsequent cancer damage, and ultimately ferroptosis without being restricted by intracellular glutathione. We conceive that this study offers a paradigm for enhancing CDT through redox dyshomeostasis and shall provide a new idea for designing COF-based nanomedicine for cancer chemodynamic therapy.

According to the above-mentioned examples and corresponding results, whether MOF- or COF-based nanomaterials are both unstable in an acidic TME and subsequently release metal ions for triggering CDT, while they are stable at neutral pH conditions. Other advantages of MOF and COF including high porosity, high molecular loading and releasing capacities, large surface area, structural/chemical diversities, and inherent biocompatibility and biodegradability have also been used for progressing CDT efficiency. Generally speaking, there are two strategies to facilitate MOF/COF-based agents: incorporating functional agents into a framework or loading functional agents into pores and channels, these ways work and are effective. Till now, more and more researches are being made in finding more suitable organic framework-based nanomaterials for CDT.

#### 3.2.3 Macromolecular nanomaterials

**Fig. 10:**
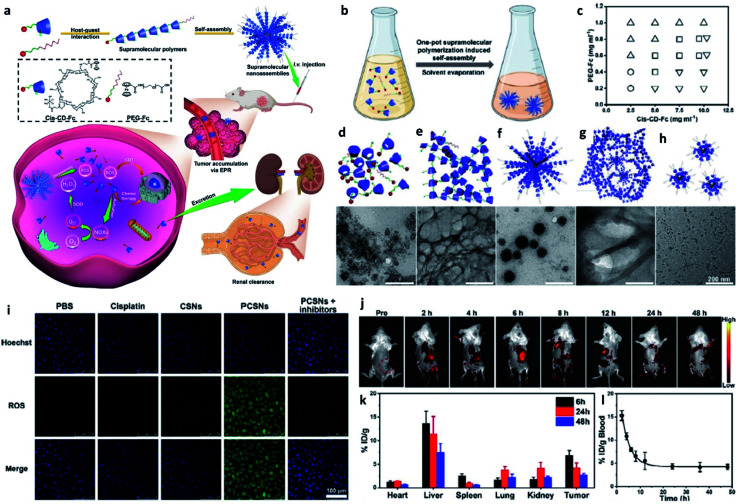
(a) Schematic synthetic process of PCSNs (a platinum(iv) nanocomplex-modified CD-Fc conjugates (cis-CD-Fc) as supramolecular monomers, CD indicates β-cyclodextrin, and SN indicates supramolecular polymer (SP) nanoparticles) for self-augmented chemo/chemodynamic cancer therapy. (b) One-pot supramolecular polymerization-induced rapid self-assembly of cis-CD-Fc monomers. (c) The product illustration for the self-assembly of SPs with varying amounts of PEGylated Fc and cis-CD-Fc monomers. Circle symbol indicates irregular aggregates, inverted triangle symbol indicates microfibers, square symbol indicates nanoparticles, and triangle symbol indicates ultrasmall nanodots. (d–h) TEM images of irregular aggregates, microfibers, nanoparticles, mixtures, ultrasmall nanodots, respectively. (i) CLSM images of 4T1 tumor cells stained by ROS probe (DCFH-DA, green). (j) Fluorescence images of 4T1 tumor-bearing mice after intravenous injection of Cy7-labelled PCSNs at different time. (k) Biodistribution of platinum-based nanoagents in the tumors and major organs at different time. (l) Blood clearance of PCSNs in 4T1 tumor-bearing mice. Reproduced with permission from ref. 56. Copyright 2021, Wiley-VCH.

It is well known that ferrocene contains two parallel cyclopentadiene rings with Fe^2+^ ions sandwiched inside has been seen as a typical Fenton agent. Besides, Ferrocene-based polymers have also been well designed with some H_2_O_2_-producing agents like ascorbic acid, glucose oxidase, and benzoyloxycinnamaldehyde to improve the production of H_2_O_2_ to make up for the shortage of endogenous H_2_O_2_ in TME, and then to convert them into toxic ˙OH for enhanced CDT. Based on the previous study, the half-life of ˙OH is short, and these produced radicals can only work on tumor cells in the immediate microenvironment of binding regions for ferrocene. However, the traditional theory about the permeability and retention (EPR) effect also tells us that the tumor penetrating requirement of a suitable size less than 30 nm, which conflicts with the size requirement of these polymers (∼100 nm). According to this, to address these restrictions, Wang's group rationally designed and developed a supramolecular nanoparticle by a simple and versatile one-step supramolecular polymerization-induced self-assembly method by using platinum(iv) nanocomplex-modified β-cyclodextrin-ferrocene (act as supramolecular monomers) (Fig. 10).^56^ Specifically, the supramolecular NP can dissociate rapidly when reacting with H_2_O_2_ in TME and then *in situ* produce ˙OH as well as platinum(iv) drug, similarly, to overcome the limitation of conventional CDT, platinum(iv) drug could be reduced into cisplatin to activate NOX for enhancing H_2_O_2_ level. Additionally, the size of the supramolecular nanoparticle is tunable and could be readily excreted from the body *via* renal clearance, thus both enhancing the EPR effect and minimizing the systemic toxicity and side effects. This example provides an idea for designing macromolecular like polymers to prepare macromolecular-based CDT agents for enhancing treatment efficiency.

Besides, high-molecular-weight and biocompatible deoxyribonucleic acid (DNA) ligands, as well as ribonucleic acid (RNA), have also been considered to use for cancer CDT. For instance, Tian's group reported a new strategy to prepare a new CDT agent that relied on DNA with high loading capacity and excellent biocompatibility, thus achieving a highly efficient CDT effect.^25^ Herein, DNA could be seen as an ideal carrier to deliver Fenton ions, such as Fe^2+^ or Mn^2+^. Specifically, these metal ions would coordinate with DNA strands at the nucleobases with high affinity or the negatively charged phosphodiester backbones. The long single-strand DNA can be condensed into nanoparticles with average diameters of about 100 nm in the presence of Mn^2+^ together with Mg^2+^ ions to yield the Mn^2+^-loading DNA nanoparticles (MDNs). The as-prepared MDNs exhibited a high carrying rate of Fenton agents (Mn^2+^) and a desirable release capacity of Mn^2+^ due to the degradation of DNA in the presence of H_2_O_2_ after being uptook in the tumor cells. Taken together, DNA-based nanocatalysts could also be considered for CDT-associated anti-cancer applications.

### 3.3 Single-atom nanozymes

Based on the theory that heterogeneous catalysis commonly occurs on the surface of a solid nanocatalyst, whose activity could be well enhanced by increasing the number of the exposed active sites. Since the intrinsic peroxidase activity of Fe_3_O_4_ NP was first reported in 2007, many nanozymes have been reported. “Nanozyme” borrows from the word “nanomaterial” and “enzyme”, meaning that some kinds of nanomaterials possess some natural enzyme activities. And most nanozymes are peroxidase mimicking and a few have catalase activity. In contrast to the enzymes and traditional artificial enzymes, nanozymes obtain advantages including high stability, low cost, mass production, and inherent multiple functionalities. These above advantages make nanozyme a promising candidate for broader applications including cancer therapy. Among many different kinds of nanozymes, single-atom catalysts (SACs) or single-atom nanozymes (SAzymes) have attracted much more attention because of their special features to achieve the maximum catalytic rate in TME. To achieve the best atomic efficiency for an enhanced specific activity, the dimensions of the active sites can be reduced by the atomic scale. Consequently, many researchers have constructed many single-atom Fenton nanozymes to trigger heterogeneous Fenton reactions with high stability, high selectivity, and sustainable activity in TME. Until now, over 7500 publications from Google Scholar have been published and more than 300 different kinds of nanomaterials with enzyme-mimicking activities for converting the substrates of oxidoreductase (*e.g.* catalase (CAT)-like, peroxidase (POD)-like, and superoxide dismutase (SOD)-like activities). Hence, developing single-atom Fenton nanozymes for CDT is also meaningful but challenging.

**Fig. 11:**
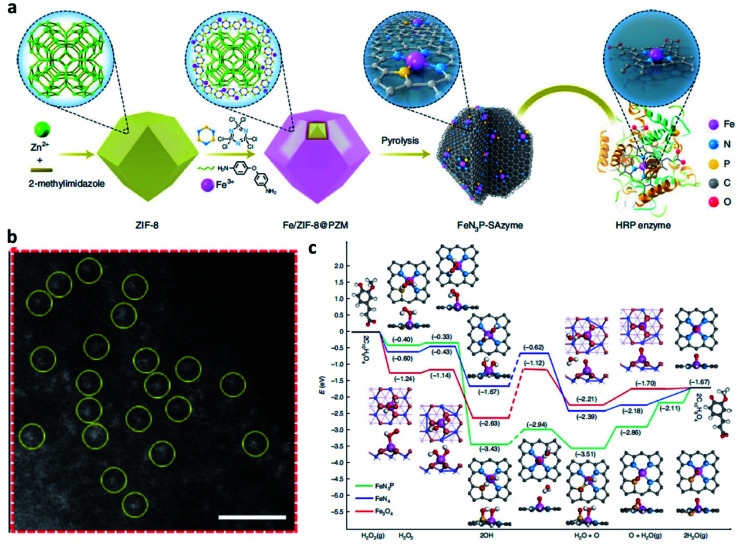
(a) Schematic synthetic process of FeN_3_P-SAzyme (SAzyme: single-atom nanozyme). (b) AC HAADF-STEM image of FeN_3_P-SAzyme. Scar bar: 1 nm. (c) DFT calculations on the POD-like activity of FeN_3_P-SAzyme, FeN_4_-SAzyme, and Fe_3_O_4_ nanozyme for comparison. Color code: C, grey; Fe, blue; N, light blue; O, light red; P, yellow; H, white. Reproduced with permission from ref. 57. Copyright 2021, Nature Publishing Group.

For example, in 2021, Yan's group reported an atomic-level engineered FeN_3_P single-atom peroxidase nanozyme called FeN_3_P-SAzyme by modulating the single-atom iron active site through precise coordination of nitrogen and phosphorus (Fig. 11a and b).^57^ The as-constructed FeN_3_P-SAzyme exhibited comparable kinetics and catalytic activity (*k*_cat_/*K*_m_ = 1.40 × 10^8^ M^−1^ min^−1^) and selectivity (*K*_m_ = 2.06 × 10^−3^ mM) to natural enzyme peroxidase (horseradish peroxidase, *k*_cat_/*K*_m_ = 1.15 × 10^8^ M^−1^ min^−1^, *K*_m_ = 5.55 mM). From the density functional theory shown in Fig. 11c, the engineered single-atom Fe active center precisely anchored by the doped P and N atoms greatly promote the catalytic kinetics of SAzyme. The single-atom P atoms and Fe sites with less positive charge as electron donors synergistically induce low barriers of surface O formation and result in fast kinetics. The three-dimensional porous structure of SAzyme simulates the role of the three-dimensional amino acid structure of enzymes, thus further enhancing the POD-like catalytic activity and substrate selectivity of SAzyme. Accordingly, with precise engineering of the geometric and electronic structure of the active site, SAzyme would play a promising breakthrough in constructing artificial nanozymes as the desirable alternative to natural enzymes for cancer therapy. In addition, the as-obtained SAzyme would also provide a bridge between homogeneous/heterogeneous catalysis and enzymatic catalysis and guidance for future design and development of nanozyme-based nanomaterials with significant prospects in clinical applications.

Another interesting work based on SAzyme is also introduced here for reference. In 2021, Lin's group proposed an innovative strategy of ferroptosis-enhanced mild photothermal therapy based on SAzyme to both maximize the efficiency of photothermal therapy and minimize damage to the healthy tissues.^58^ Specifically, the as-prepared Pd-doped SAzyme with atom-economic utilization of the active centers shows both POD-like and glutathione oxidase (GSHox)-like activities and high photothermal conversion performance, thus resulting in ferroptosis inducing the up-regulation of reactive oxygen species (ROS) and LPO (lipid peroxides). The accumulation of ROS and LPO offers a useful approach for cleaving heat shock proteins, finally enabling Pd SAzyme-triggered mild-temperature photothermal therapy. Therefore, this work provides an attractive paradigm of ferroptosis improving mild photothermal therapy at a safe temperature for highly efficient tumor therapy, and firstly introduce the concept of ferroptosis-boosted photothermal therapy based on SAzyme, thus offering another hopeful direction for future cancer chemodynamic therapy according to single-atom Fenton nanozymes.

Overall, we believe that single-atom nanozyme containing both inherent physical–chemical features of nanomaterials and enzyme-like catalytic capacities could significantly improve the development of CDT agents for specific and high-efficient cancer chemodynamic therapy. Moreover, nanozymes would also boost the crosstalk between biology and nanotechnology, thus bringing novel insights and academic enthusiasm.

### 3.4 Electron-rich substance hybrid nanomaterials

**Fig. 12:**
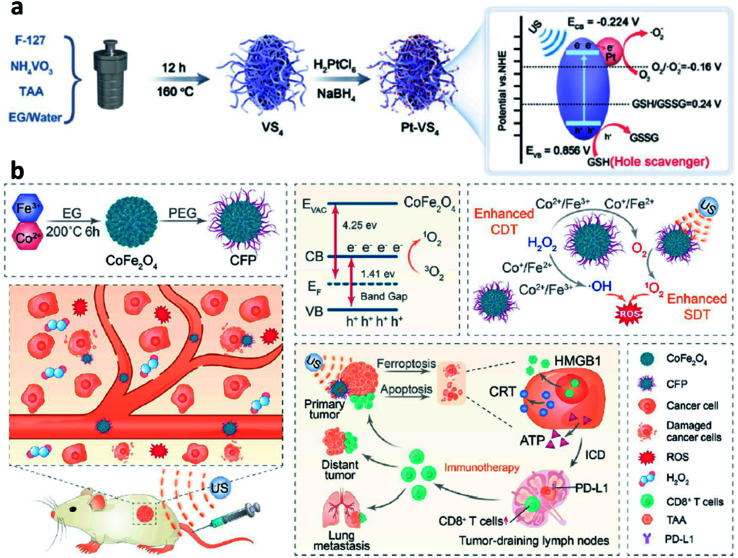
(a) Schematic synthetic process of Pt–VS_4_ for enhanced chemodynamic therapy. Reproduced with permission from ref. 59. Copyright 2021, Wiley-VCH. (b) Schematic synthetic process of CFP (C: Co; F: Fe; P: PEG) and its working mechanism for boosting chemodynamic therapy with the elicitation of a robust immune response. Reproduced with permission from ref. 60. Copyright 2021, American Chemical Society.

According to the above-mentioned mechanism, the cycling of the redox couple like Fe^3+^/Fe^2+^ is important for the Fenton/Fenton-like reaction. Hence, developing strategies to enhance the localized density of electrons and accelerate the cycling of redox couples would improve the utilization of H_2_O_2_ and broaden the pH range, thus enhancing the overall reaction efficiency of the Fenton reaction. Encouraged by the study between electrons and Fenton chemistry (*e.g.*, from semiconductors, electron-rich nanomaterials, and plasmonic materials) in the field of water treatment, employing electron-rich substance hybrid nanomaterials in CDT could also be a promising choice to steadily favor the conversion from H_2_O_2_ to ˙OH. For instance, in 2021, Lin's group proposed a branched vanadium tetrasulfide nanodendrites that contained a narrow bandgap for elevating intratumoral ROS in the current CDT, which allows a more effortless separation of electron–hole pairs for ROS production (Fig. 12a).^59^ In specific, noble metal platinum (Pt) NPs and endogenous overexpressed GSH in TME are rationally engineered to maximum its sono-sensitized effect. Herein, Pt as a co-catalyst can be used to trap electrons, while GSH as a hole scavenger can effectively capture holes. In contrast to the pristine VS_4_ nanodendrites, the as-prepared GSH–Pt–VS_4_ nanocomposite can significantly prolong the lifetime of the free charge and confer a more efficient ROS generation ability. In addition, this nanoplatform can remodel the TME to realize ROS overproduction, which could be attributed to the reason that electrons would enrich on the conduction band of VS_4_ for greatly reducing the reaction potential of O_2_/˙O_2_^−^ (the source for generation ROS or H_2_O_2_*via* one- or two-electron reduction reaction). Meanwhile, the GSH would be oxidized by the produced holes from GSH–Pt–VS_4_ nanocomposite to amplify the intratumoral oxidative stress. The localized concentration of electrons plays the most important role in improving catalytic efficiency. This work proposes an electron-rich hybrid nanomaterial by doping noble metal on the surface of semiconductors to form an electron-focusing site for reducing the reaction potential of O_2_/˙O_2_^−^ for enhanced catalytic efficiency and proves that nanomaterials can be optimized by the charge separation engineering method. We conceive that this nanoplatform provides another direction for developing novel CDT agents with electron-rich levels to greatly reduce the reaction potential.

Another necessary example could be also listed in this section for reference based on bimetal electron-rich nanomaterials. In 2021, Xue's group reported PEGylated CoFe_2_O_4_ nanoflowers with multiple enzyme-like activities, which can act as a bioreactor that responses to TME cues and be prepared by a typical solvothermal method for boosting CDT (Fig. 12b).^60^ Notably, along with the CDT treatment process, an elicitation of robust immune response occurred. And the CFP nanoflower containing multivalent elements (Fe^2+^/^3+^, Co^2+^/^3+^) show highly Fenton-like and catalytic activity. From another angle, CFP nanoflower itself with a narrowed bandgap possesses desirable treatment performance that can be attributed to the rapid separation of electron–hole pair. After efficient accumulation in tumor tissues, CPF could generate a large number of cytotoxic ˙OH relying on the enhanced Fenton-like reactions. More importantly, the generated ROS can then efficiently trigger immunogenic cell death owing to the effective elicitation of anticancer immunity with the assistance of an immune checkpoint blockade. Consequently, this paradigm proposes meaningful insights for constructing electron-rich nanocomposites with a narrow bandgap for cancer theranostics.

In conclusion, benefiting from the significant development between nanomedicine and Fenton chemistry, various kinds of Fenton agents with CDT therapeutic efficiency have been developed. And these as-prepared agents also perform desirable physical–chemical properties and some of them have also been well applied in the practical treatment with the mice model for cancer therapy. However, the current selection of therapeutic agents is still more oriented towards the inorganic therapeutic agents, and there is also a lack of research on *in vivo* mechanisms of action, metabolic pathways, and physiological toxicity of inorganic agents. For the selection of therapeutic agents, we prefer a more balanced choice rather than just focusing on their efficacy and ignoring their side effects.

## 4. The guidance for improving CDT performance

After the introduction of the selection of the various Fenton agents for improving the efficiency of chemodynamic therapy, this section would provide detailed guidance for improving CDT performance from other aspects. It could be divided into the following parts: Section 4.1 Distinguished from the above-mentioned mainstream Fenton agents, developing novel Fenton agents to breakthrough their inherent drawbacks; Section 4.2 remodeling TME for a more suitable reaction environment (lowing pH, increasing H_2_O_2_ content, and decreasing GSH level); Section 4.3 introducing external stimulation for enhanced electron transfer (photo-, ultrasonic-, thermal-, electric-, and magnetic-induced) and combing other treatment methods guided by the advanced imaging technologies.

### 4.1 Developing innovative Fenton agents

To address the inherent drawbacks of current Fentons agents, developing innovative Fenton therapeutic agents that are distinguished from the conventional Fenton agents is necessary but challenging. In this section, we would provide several attempts as examples and make a discussion to point a direction for designing novel Fenton agents.

**Fig. 13:**
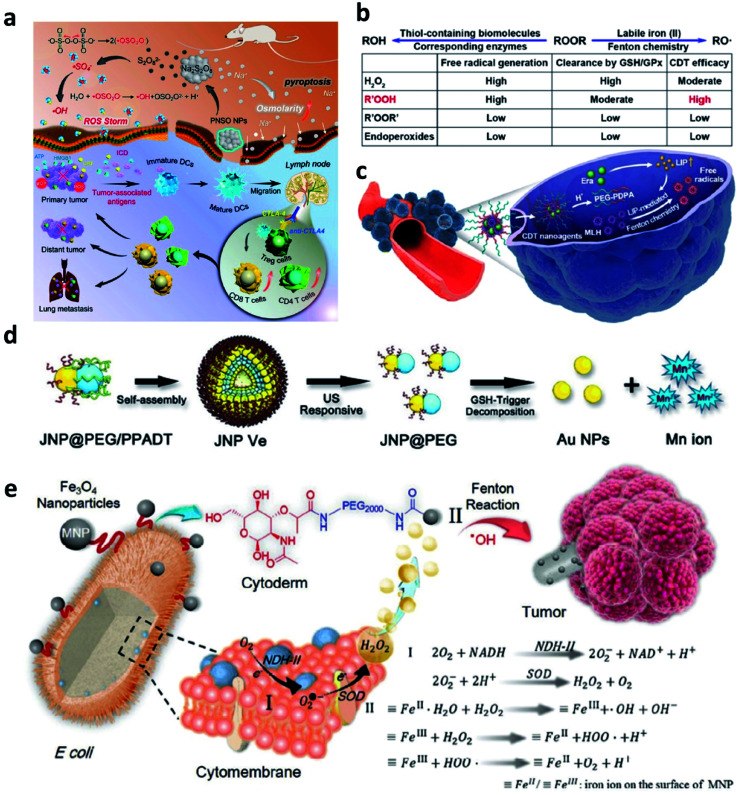
(a) Schematic illustration of the therapeutic mechanism of PNSO nanoparticles (PNSO: peroxydisulfate nanoparticles modified with PEGylated and lecithos). Reproduced with permission from ref. 61. Copyright 2020, American Chemical Society. (b and c) Schematic illustration of factors affecting labile iron pool-mediated chemodynamic therapy efficacy of ROOR and the utilize of methylinoleate hydroperoxide-carrying nanoagents for enhanced chemodynamic therapy (era: erastin). Reproduced with permission from ref. 62. Copyright 2020, American Chemical Society. (d) Schematic synthetic process of amphiphilic Janus Au–Mn nanoparticles into multi-functional vesicles *via* self-assembly. Reproduced with permission from ref. 63. Copyright 2020, Wiley-VCH. (e) Schematic illustration of bacteria-based bioreactor for cancer chemodynamic therapy. Reproduced with permission from ref. 65. Copyright 2019, Wiley-VCH.

Firstly, to break the limitation of ROS generation rate in TME because of the relatively low concentration of H_2_O_2_, strict Fenton reaction conditions like pH range from 3 to 4, and so on. In 2020, Zhang's group pioneeringly and firstly explored a new agent with highly efficient ROS generation ability. Specifically, they successfully prepared phospholipid-modified Na_2_S_2_O_8_ nanoparticles as new ROS production agents for cancer treatment (Fig. 13a).^61^ After gradual degradation in the tumor sites, Na^+^ and S_2_O_8_^2−^ would then be released to take part in reaction to generate cytotoxic ˙SO_4_^−^ (sever as a new type of ROS) and ˙OH instead of considering the amount of H_2_O_2_ content and pH value in TME. In addition, PNSO nanoparticles can easily accumulate in the tumor cells and produce a large number of Na^+^ into tumor cells, thus resulting in a surge of osmolarity for the rapid cell rupture and lysis. The unusual manner of cell death named caspase-1-related pyroptosis can be induced by the generation of osmotic pressure. This work is creative and exhibits a possibility to develop some Fenton agents that are not limited by the TME, and we conceive that this work points a promising direction to develop novel Fenton agents to generate novel reported ROS like ˙SO_4_^−^. And this work would significantly broaden the thinking of the exploring of alternative anticancer nanodrugs.

Another work about developing a new strategy to enhance CDT efficiency is also needed to introduce. To solve the issue that introducing an excess amount of exogenous Fenton heavy metals may unavoidably bring potential side effects to normal tissues like acute and chronic damages. In 2020, Chen's group reported a novel CDT strategy that creatively uses the intracellular labile iron pool (LIP, which acts as the endogenous Fenton-active metals) for activating the Fenton reaction to generate ˙OH and hydroperoxides (R′OOH) for killing cancer cells (Fig. 13b and c).^62^ Similar to the ˙SO_4_^−^, R′OOH is also another new type of ROS that obtaining strong oxidase activity for inducing cell death. The LIP-initiated nontoxic-to-toxic conversion of R′OOH along with increasing LIP content in tumor cells, enabling highly efficient and specific CDT. Moreover, the cascade responsive nanodrugs comprising encapsulated methyl linoleate hydroperoxide and LIP-boosting agents in pH-responsive polymer compounds were also constructed for enhanced CDT. Taken together, this study not only provides another way to use endogenous Fenton agents for cancer chemodynamic therapy but also paves a paradigm for the exploration of CDT agents with high catalytic performance activated by intracellular LIP.

To realize the multifunctionalities of CDT agents to achieve some specific goals, a special nanomaterial named by Janus (god has two faces) has emerged for cancer therapy with excellent catalytic activities. For example, in 2020, Yang's group paved an ultrasound and GSH dual responsive Au–MnO Janus vesicles modified by PEG and a ROS-sensitive polymer (Fig. 13d).^63^ Under the ultrasound irradiation, Au–MnO Janus vesicles could rapidly decompose into Au–MnO nanoparticles and then reacted with GSH to generate Mn^2+^ for CDT and Au nanoparticles to sever as numerous cavitation nucleation sites, finally synergistically enhancing the ROS generation. Yang's group also reported an X-ray and GSH dual responsive Janus vesicles encapsulating the near-infrared fluorescence dye (IR1061).^64^ When reacting with GSH, the as-obtained Janus vesicles would also rapidly dissociate into smaller nanoparticles and release Fenton manganese (Mn^2+^) for cancer chemodynamic therapy. The combination of Janus vesicles and X-ray irradiation achieve a synergistic and enhanced treatment efficiency (1 + 1 > 2). Applying multiple compounds into one nanoparticle not only enhances the catalytic rate but also introduces other treatment methods easily and feasibly.

Apart from these above-mentioned nanomaterials, in 2019, Zhang's group provided interesting research based on bacteria (Fig. 13e).^65^ As we know, synthetic biology based on microorganisms including engineered bacteria has been well employed in anticancer therapy and exhibits desirable performance. Herein, engineered bacterium *Escherichia coli* (*E. coli*) MG1655 was designed to combine NDH-2 enzyme (respiratory chain enzyme II) overexpression (Ec-pE). And engineered *E. coli* could colonize at tumor sites and significantly increase the localized concentration of H_2_O_2_. With its powerful proliferation rate, the H_2_O_2_ content at tumor sites is expected to be effectively replenished within a short period, and this replenishment process is sustainable. Subsequently, Fe_3_O_4_ nanoparticles as the typical Fenton agents were covalently linked to *E. coli* to induce Fenton reactions after accumulated in TME. This work provides biology nanomaterials based on bacteria that can realize effective tumor colonization and achieve an H_2_O_2_ self-supply sustainable chemodynamic therapy without additional H_2_O_2_ provision.

### 4.2 Remodeling TME for optimizing CDT

Based on the above-mentioned mechanism about Fenton chemistry, the Fenton reaction is significantly influenced not only by the catalytic ions released from Fenton agents but also by the reaction condition, mainly including the pH range and the amount of H_2_O_2_ (reaction substrate) and GSH (major antioxidants in TME). Hence, regulating TME for enhancing the efficiency of CDT is another effective manner and has attracted much attention.

#### 4.2.1 *In situ* adjusting pH range

For the Fenton chemistry, a suitable reaction pH range seems necessary and important because a relatively low pH environment not only prevents the precipitation of ferrous and iron ions but also improves H_2_O_2_ degradation. For TME, the pH range commonly maintains from 6.5 to 7.0, and the endosomes of cancer cells have a lower pH of ∼5.0, and lysosomes of cancer cells have a pH of ∼4.5. For most reported nanomaterials used for CDT, the amount of released metal ion is restricted by the requirement for a mildly acidic environment, thus inhibiting the Fenton reactions. Therefore, *in situ* lowing the pH value of TME or delivering these Fenton agents to the nucleus or lysosomes may be game-changers. Additionally, some other alternative strategies have also been proposed to decrease the pH value of TME including gene silencing and biochemistry reactions.

**Fig. 14:**
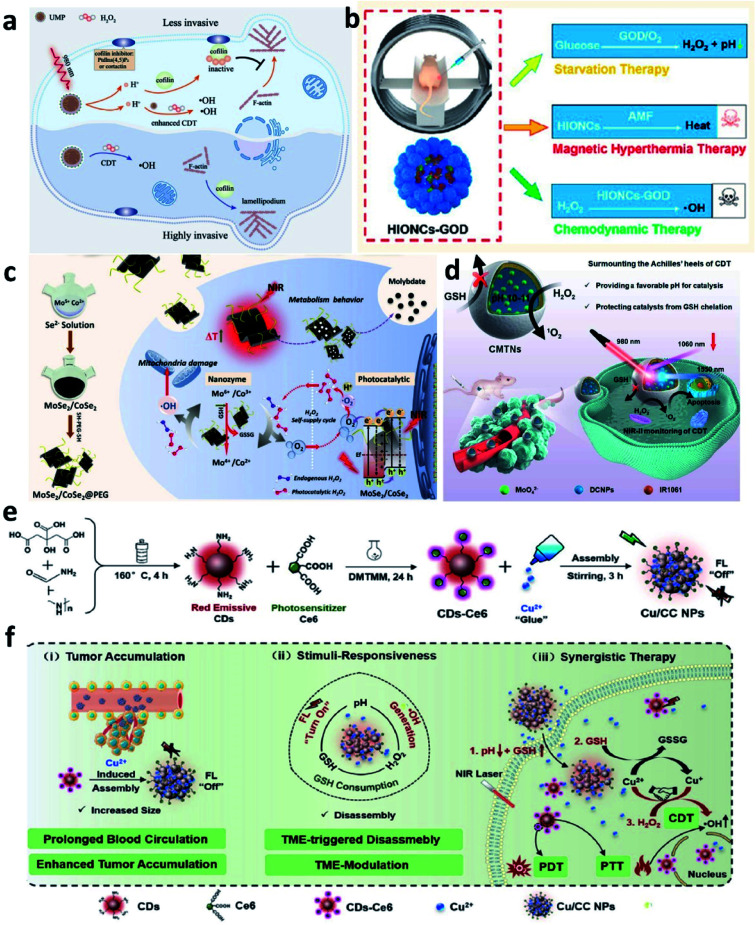
(a) Schematic illustration of the therapeutic mechanism of inhibiting tumor invasiveness and enhancing CDT by increasing H^+^ content in tumor cells. Reproduced with permission from ref. 66. Copyright 2021, Wiley-VCH. (b) Schematic illustration of the combined starvation therapy, magnetic hyperthermia therapy, and chemodynamic therapy. Reproduced with permission from ref. 67. Copyright 2020, American Chemical Society. (c) Schematic illustration of PEGylated MoSe_2_/CoSe_2_ nanoparticles for photothermal/chemodynamic therapy with self-supplying H_2_O_2_*via* photocatalysis. Reproduced with permission from ref. 68. Copyright 2020, Wiley-VCH. (d) Schematic illustration of CMTNs (catalytic microenvironment-tailored nanoreactor) for enhanced CDT by providing favorable pH and protecting catalysts from GSH chelation. Reproduced with permission from ref. 69. Copyright 2020, Wiley-VCH. (e and f) Schematic synthetic process of Cu/CC nanoassemblies and its application of cancer therapy. Reproduced with permission from ref. 70. Copyright 2020, Wiley-VCH.

For example, in 2021, Bu's group reported a near-infrared laser-triggered nanoscale H^+^ supplier composed of upconversion nanoparticles (core) and MIL-88B (shell, for interior photoacids loading) (Fig. 14a).^66^ After accumulation in tumor cells *via* the EPR effect, the photoacids loading can improve H^+^ transients in tumor cells, thus converting the cofilin to an inactive state. Inhibiting cofilin activity can effectively induce defects in directional lamellipodia formation and the locomotory ability of cell invasion, finally benefiting antimetastatic therapy. In addition, the released iron ions that serving as catalytic active sites in MIL-88B would exhibit an enhanced catalytic activity owing to the increasing content of H^+^ in TME. This creative work offers new insights into lowing pH in tumor cells, which could not only improve chemodynamic therapy but also regulate cofilin protonation for enhancing antimetastatic therapy. In addition, in 2020, Lin's group developed a magnetic nanocatalyst composed of glucose oxidase-loaded iron oxide nanoplatform to perform synergistic therapy (starvation-chemodynamic-hyperthermia combined therapy) (Fig. 14b).^67^ This work can be seen as a representative of many efforts, in a nutshell, the reason for the decrease of pH in TME can be attributed to the employment of the glucose oxidase. Until now glucose oxidase has been widely applied in cancer therapy because of its excellent enzyme activity for converting O_2_ into H_2_O_2_ along with the generation of H^+^, which greatly matches the requirement of optimal chemodynamic therapy. We conceive that glucose oxidase would have a further application after the limitations have been well solved including easy inactivation, premature leakage during transport, and so on. Herein, we just list only two common strategies to efficiently and consistently lower pH at the tumor sites and ultimately enhance CDT efficacy. In the future, considering the long-term development and further clinical application, we think that developing novel Fenton nanomaterials without depending on acidic conditions shall be another direction of development.

#### 4.2.2 *In situ* increasing H_2_O_2_

Although H_2_O_2_ is overexpressed in tumor cells compared with normal cells proved by previous studies, the amount of H_2_O_2_ in TME is still not enough for triggering the maximum treatment efficiency of chemodynamic therapy. Therefore, the strategy for *in situ* increasing the H_2_O_2_ content is an effective and straight manner to significantly promote the catalytic efficiency in tumor regions. And studies on increasing the H_2_O_2_ content in TME by performing endogenous reactions and/or delivering H_2_O_2_-loading nanocarriers at the tumor sites have been well developed. For instance, to accelerate the Fenton reaction, the above-mentioned natural glucose oxidase enzymes or artificial glucose oxidase-like nanozymes that can catalyze O_2_ into H_2_O_2_ have been successfully employed in enhancing cancer chemodynamic therapy by many research groups. Apart from these materials with enzyme activities, in 2020, Qu's group successfully prepared a PEGylated MoSe_2_/CoSe_2_ (MoSe_2_/CoSe_2_@PEG) nanosheet to break through the restriction of limited intratumoral H_2_O_2_ content (Fig. 14c).^68^ Notably, different from glucose oxidase, MoSe_2_/CoSe_2_@PEG nanosheet can react with O_2_ under the activation of near-infrared laser irradiation *via* a two-electron reduction reaction. In detail, the dissolved oxygen would be firstly be reduced to ˙O_2_^−^ by the photoexcited electrons from the conduction bands of the nanosheet. Then, ˙O_2_^−^ would be further reduced to H_2_O_2_ to make up for the shortage of endogenous H_2_O_2_. The effective replenishment of H_2_O_2_ finally enhances the treatment efficiency of chemodynamic therapy. This work successfully applies the photocatalytic effect into tumor cells, which makes the supply of H_2_O_2_ is controllable and on-demand.

Whether glucose oxidase enzymes or photocatalysts, the reaction process both needs the participation of oxygen. However, the concentration of O_2_ in tumors is also extremely limited, thus hindering the further development of the use of glucose oxidase and photocatalysts. Consequently, other strategies that do not rely on the participation of O_2_ shall be considered. For example, some strategies for relieving tumor hypoxia could be combined, such as delivering O_2_-loading nanocarriers into tumor cells to supplementary, employing some enzymes with catalase activities, gene slicing technology, and so on.

#### 4.2.3 *In situ* depleting GSH

It is well known that cells could protect themselves from the damage by the generated highly reactive and cytotoxic ROS *via* a series of redox reactions. This protection process is achieved by the cellular antioxidant scavenger systems that are composed of enzymes (including scavenger enzymes: glutathione peroxidase, catalase, and superoxide dismutase), and some reducing substances (vitamin C, cysteine, and glutathione) to balance the ROS within a normal level. Notably, similar to the overexpressed H_2_O_2_, many studies have evidenced that the content of GSH is also greatly elevated in many kinds of tumor cells, thus endowing tumors with strong resistance to these ROS-mediated traditional anticancer methods including chemodynamic therapy. Hence, depleting the elevated GSH in TME should be made great efforts to realize to further boost CDT. For example, in 2021, Chen's group reported a catalytic microenvironment-tailored nanoreactor named CMTN that is composed of MoO_4_^2−^ nanocatalysts and alkaline sodium carbonate within liposomes (Fig. 14d).^69^ CMTN could not only provide a favorable pH range for MoO_4_^2−^ induced generation of ROS but also protect MoO_4_^2−^ from being removed by GSH because of the impermeability of liposomal lipid membrane to GSH. This work provides a new concept of “protection” instead of “depletion”, which may point out a new insight for designing antioxidant agents based on the concept of protection. Besides, in 2020, Lin's group proposed a way to prepare TME stimuli-responsive nanoparticles for synergistic cancer therapy *via* combining Fenton ion Cu^2+^ and photosensitizer (chlorine e6, Ce6) modified carbon dots (CDs-Ce6) (Fig. 14e and f).^70^ After entering and accumulating in the tumor cells, the nanocomposites named Cu/CC NPs would exhibit a strong ability of ROS generation. The released Cu^2+^ could firstly react with GSH to produce GSSG and Cu^+^, and the generated Cu^+^ would then start the Fenton reaction to produce ˙OH. It is not difficult to find that Cu^2+^ not only depletes GSH in TME to amplify the intracellular oxidative stress *via* a redox reaction but also induces the occurrence of Fenton reaction for enhanced CDT.

*In situ* H_2_O_2_ generation for enhanced oxidative stress and GSH depletion for decreased reduction resistance is effective manners for promoting the efficiency of CDT. To our knowledge, current studies only pay attention to extremely limited substances including H_2_O_2_ and GSH. There is plenty of room for scientists to explore next-generation Fenton agents that could react with other substances like catalase, superoxide dismutase, vitamin C, and so on.

### 4.3 External stimulation-assisted Fenton reactions

Introducing external stimulation for enhanced electron transfer (photo-, ultrasonic-, magnetic-, thermal-, and electric-induced) and combing other treatment methods can effectively enhance the catalytic activity of both heterogeneous and homogeneous Fenton/Fenton-like reactions. Hence, external stimulations could also be applied in CDT, thus not only optimizing the CDT performance but also combing other therapeutic manners including photodynamic, sonodynamic, photothermal, electrodynamic, magnetic hyperthermia therapies, and so on. In this section, recent research progress based on external stimulation-assisted Fenton agents is provided for discussion to underly the detailed mechanisms for enhanced CDT activities. Additionally, following the introduction of external stimulation, the corresponding imaging methods for monitoring, guiding, and assessing CDT are also added for discussion.

#### 4.3.1 Photo-assisted Fenton reactions

Photo-induced therapies including photodynamic therapy (PDT) and photothermal therapy (PTT) and photo-excited imaging methods like photoacoustic imaging (PAI), photothermal imaging (PAI), and fluorescence imaging (FLI) have been widely employed in the field of phototherapy. And various photo-triggered nanomaterials have also been well prepared with many attractive features, such as high ROS production rate, fluorescence quantum yield, and photothermal conversion efficiency. Because the wavelength and intensity of the using laser are controllable and adjustable, on-demand phototherapy could be easy to realize. Accordingly, noninvasive phototherapy has become a promising treatment alternative very recently. Interestingly, much research proves that photo-induced energy could also accelerate the Fenton reaction kinetics based on thermodynamic theory. Essentially, the enhanced efficiency of Fenton reactions could be attributed to the accelerated transfer between electrons after laser irradiation, thus reducing the reaction potential between H_2_O_2_ and ˙OH. Therefore, introducing the photo-Fenton agents in CDT is also a promising strategy for effectively promoting reaction efficiency. Till now, various photo-Fenton nanocatalysts have been well developed for performing photodynamic, photothermal, and/or chemodynamic combined therapy guided by PAI and other advanced imaging methods. Next, for the selection of the wavelength of the using laser, NIR laser would be a tendency in the future because of its deeper penetration compared with UV/vis light for practical applications. Furthermore, compared with the commonly used 808 nm laser in NIR-I (700–900 nm), 1064 nm laser in NIR-II (1000–1700 nm) used in this study is more desirable due to its higher maximum permissible exposure (1 *vs.* 0.33 W cm^−2^) and deeper tissue penetration depth. Taken together, photo-Fenton nanocatalysts that could be excited by the NIR-II laser could be seen as the most promising direction to our knowledge.

**Fig. 15:**
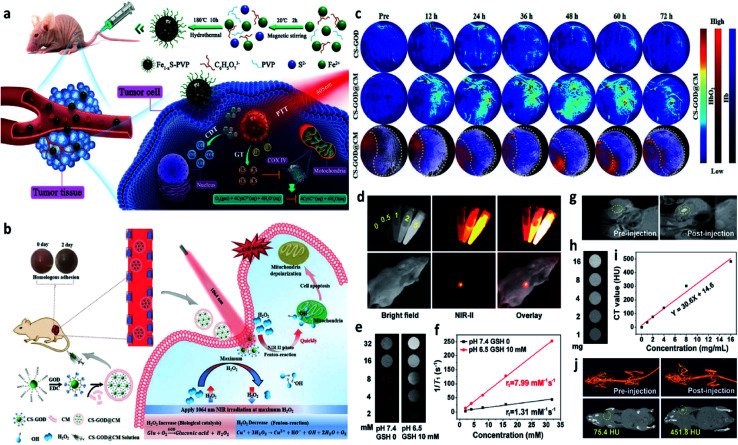
(a) Schematic illustration of the Fe_1−*x*_S-PVP-mediated gas/photothermal/chemodynamic synergistic therapy. Reproduced with permission from ref. 71. Copyright 2021, Wiley-VCH. (b) Schematic illustration of the enhanced Fenton reaction irradiated by NIR-II laser for the treatment of breast cancer. (c) PA images of tumors from 4T1 tumor-bearing mice treated with or without CS-GOD/CS-GOD@CM nanoparticles at different time points. (CS: Cu_2−*x*_Se, CM: tumor cell membrane). Reproduced with permission from ref. 72. Copyright 2020, Wiley-VCH. (d) *In vitro* FL images of PEG/LDNPs@CMSNs (copper/manganese silicate nanosphere (CMSN)-coated lanthanide-doped nanoparticles (LDNPs)) containing different concentrations and *in vivo* images of tumor-bearing mice under 980 nm laser irradiation. (e and f) *In vitro* T_1_-weighted MR (magnetic resonance) images of PEG/LDNPs@CMSNs and PBS and the corresponding relaxation rate *r*_1_*versus* concentration. (g) *In vivo* T_1_-weighted MR images of tumor-bearing mice treated with or without PEG/LDNPs@CMSNs. (h–i) *In vitro* and *in vivo* CT (computed tomography) images. Reproduced with permission from ref. 73. Copyright 2020, American Chemical Society.

For example, in 2021, Chen's group reported nanoparticles that were composed of polyvinyl pyrrolidone modified iron sulfide (Fe_1−*x*_S-PVP NPs) *via* a typically one-step hydrothermal method (Fig. 15a).^71^ When under the NIR-I laser irradiation at 808 nm, Fe_1−*x*_S-PVP NPs show a high photothermal conversion efficiency (24%), which means that photo-energy could effectively be translated into heat in the localized tumor sites. Then, the rapidly released heat would further facilitate the Fenton reaction to produce abundant ˙OH for CDT. Meanwhile, the photothermal would also induce the generation of H_2_S gas for gas therapy, which could suppress the activity of the enzyme (COX IV, cytochrome c oxidase) in tumor cells, thus inhibiting the growth of tumors. This work proves that the introduction of photo-energy could not only produce heat for enhancing CDT but also realize a gas therapy in one nanoplatform. Another work that should be listed here because it is a typical example to show us the advantage of applying NIR-II laser to boost the Fenton reaction kinetics. In 2020, Gao's group proposed a biomimetic CS-GOD@CM (composed of ultra-small Cu_2−*x*_Se, glucose oxidase, and tumor cell membrane) nanocatalysts for NIR-II laser enhanced CDT of breast cancer (Fig. 15b).^72^ The Fenton reaction could be boosted by the reproduction of H_2_O_2_*via* the glucose oxidase's enzyme catalytic effect and the effective activation by the NIR-II laser irradiation at 1064 nm, thus maximizing the generation of ˙OH. All these treatment processes were under the monitor of photoacoustic imaging, through these images, the best treatment point could be easy to select. As evidenced in Fig. 15c, CS-GOD@CM can be retained in tumors for more than two days with the protection of tumor cell membrane and produce a ∼2.6 fold in H_2_O_2_ to enhance the Fenton reaction activity exposed to the NIR-II laser irradiation. This work firstly demonstrates the efficiency could be significantly enhanced by the NIR-II light guided by photoacoustic imaging. Apart from photoacoustic imaging, other imaging technologies, such as fluorescence imaging, magnetic resonance imaging, and computed tomography imaging, could also guide the treatment process with high resolution as exhibited in Fig. 15d–j.^73^ Surprisingly, multiple imaging methods are centralized in one nanocatalyst without introducing other functional compounds, which is greatly beneficial to the clinical application. Additionally, high-energy X-rays with wavelengths of 0.001–10 nm can efficiently deliver a dose to the tumor sites, sparing the surrounding healthy tissues. Hence, X-ray is also an effective external physical excitation, which could be considered in the future under the premise of ensuring safety. In 2021, Yang's group presented an attempt based on X-ray enhanced CDT,^64^ these experimental results are interesting and attractive, but the development in this field is still lack, further exploration remains urgently needed.

#### 4.3.2 Sono-assisted Fenton reactions

**Fig. 16:**
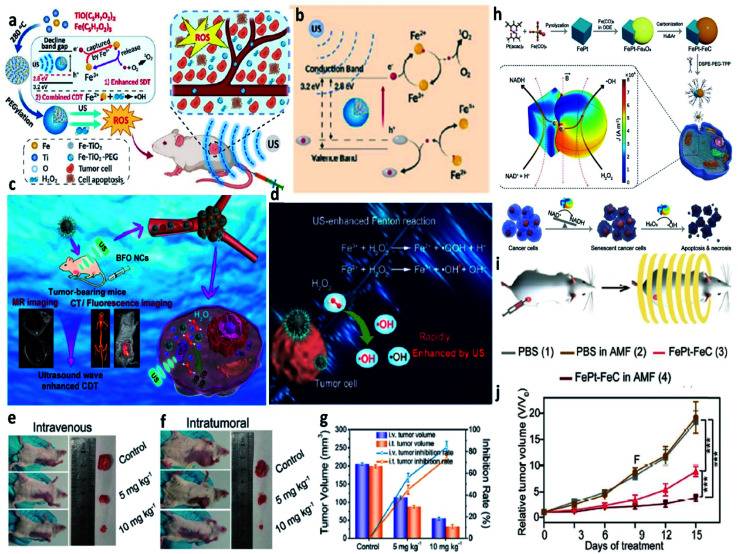
(a) Schematic synthetic process of PEGylated Fe-TiO_2_ (Fe-TiO_2_-PEG) nanoparticles for ultrasound-enhanced CDT. (b) Schematic illustration of the therapeutic mechanisms of Fe-TiO_2_-PEG nanoparticles under ultrasound irradiation. Reproduced with permission from ref. 74. Copyright 2020, American Chemical Society. (c) Schematic illustration of BFO (B: Bi, F: Fe) nanocatalysts for ultrasound-enhanced cancer chemodynamic therapy guided by multiple imaging methods. (d) Schematic comparison of the typical Fenton reaction and ultrasound-enhanced Fenton reactions. (e and f) Representative photographs of tumors for intravenous and intratumoral therapeutic manners, respectively. (g) Tumor inhibition rates and tumor volumes from different groups. Reproduced with permission from ref. 75. Copyright 2020, American Chemical Society. (h) Schematic synthetic process of FePt–FeC heterostructures and the schematic illustration of the therapeutic mechanisms of FePt–FeC@TPP (TPP: DSPE-PEG-TPP) for magneto-electrocatalytic-assisted chemodynamic therapy. (i) Schematic illustration of the treatment process. (j) Relative tumor volume curves of tumors from 4T1 tumor-bearing mice treated with different groups. Reproduced with permission from ref. 76. Copyright 2021, Wiley-VCH.

Different from the introduction of light, ultrasound (∼20 kHz, safe wave) is another type of energy and possesses a deeper penetration depth compared with both NIR-I and NIR-II lasers. More importantly, ultrasound has been widely applied in clinics for disease diagnosis and treatment, such as B-scan ultrasonography. With the rapid development of medical imaging, ultrasound has been concerned and applied in the field of cancer treatment. This is based on the understanding of the working mechanism of ultrasound that ultrasound-induced energy is beneficial for local tumor ablation, as well as for enhancing the catalytic activity of the Fenton reaction. For instance, in 2020, Liu's group successfully synthesized novel chemo/sonosensitizers composed of PEGylated ultrasmall iron-doped titanium oxide nanodots (Fe-TiO_2_-PEG NDs) for sonodynamic and chemodynamic combined therapy (Fig. 16a and b).^74^ Fe-TiO_2_-PEG NDs could not only promote the ultrasound-triggered ROS generation for sonodynamic therapy but also boosting the release of NDs to generate ˙OH for enhanced chemodynamic therapy. Owing to the combination of CDT and SDT, Fe-TiO_2_-PEG NDs could achieve a desirable tumor inhibition rate both *in vitro* and *in vivo*. This work presents a novel type of multifunctional sonosensitizer by doping TiO_2_ with metal ions for highly efficient cancer chemodynamic and sonodynamic therapy. Besides, to conquer the restrictions covering limited skin penetration depth and unavoidable phototoxicity in photoinduced therapies, in 2020, Zhao's group provided bismuth ferrite nanocatalysts (BFO NCs) for ultrasound-enhanced CDT of malignant tumors guided by multiple imagings (Fig. 16c–g).^75^ As expected, the BFO NCs exhibit highly efficient ultrasound-enhanced production of ˙OH, which is attributed to the cavitation bubbles generated by ultrasound that trigger partial grievous turbulence and improve the transfer rate of the Fenton agents. Both *in vitro* and *in vivo* results demonstrate that BFO NCs perform effective inhibition of tumor growth under ultrasound irradiation, and the treatment process is monitored by multiple imagings. Hence, this work shows a promising sono-assisted Fenton agent for noninvasive and efficient chemodynamic therapy.

#### 4.3.3 Magnetic-assisted Fenton reactions

Similar to the sono-assisted Fenton agents, magnetic-assisted Fenton-based therapy has been performed in the field of cancer therapy owing to its unlimited penetration and negligible adverse effects on healthy tissues. For instance, in 2021, to break through the hinders of the low efficiency of intracellular catalysis, Bu's group developed a magneto-electronic manner for highly efficient intracellular catalytic reaction by employing FePt–FeC heterostructures in mild alternating magnetic fields (96 kHz and *B* ≤ 70 mT) (Fig. 16h–j).^76^ The results of finite element simulation demonstrate a high density of charge gathering between the interface of FePt–FeC under the magnetic field. Subsequently, the enhanced catalytic reaction of FePt–FeC under the magnetic stimulation would significantly reduce the β-nicotinamide adenine dinucleotide enzyme in cancer cells for facilitating further treatment. This work demonstrates that cancer cells could be efficiently killed by the CDT based on the magnetic-enhanced Fenton reactions.

#### 4.3.4 Thermal-, and electric-assisted Fenton reactions

Apart from the abovementioned three mainstream external energy fields, thermal and electronic fields have also been reported to improve the treatment efficiency of CDT. But the development of thermal- and electric-assisted Fenton reactions goes far behind than photo-, sono- and magnetic-assisted Fenton reactions due to the requirement for the *in vivo* application for thermal and electronic is extremely strict to ensure biosafety. For instance, in 2017, Bu's group synthesized FeS_2_-PEG NP to induce localized heat for accelerating and enhancing the Fenton reaction by Fe^2+^, finally amplifying the catalytic efficiency of CDT.^77^ Impressively, the FeS_2_-PEG NP exhibited sufficient performance in ˙OH generation when the localized heat was introduced. From the data captured after the administration of various treatments, FeS_2_-PEG NP assisted by heat could significantly suppress tumor growth due to the heat-enhanced CDT. For the electric-assisted Fenton reactions, in 2021, Li's group reported Fe_3_O_4_@Pt NP for combining both electrodynamic therapy (EDT) and chemodynamic therapy.^78^ Interestingly, Fe_3_O_4_@Pt NP could effectively induce ˙OH production based on the catalytic reaction on the surface of Pt NP triggered by an electric field. Subsequently, it may improve the catalyzation of H_2_O_2_*via* electric-enhanced Fenton reaction. Consequently, considerable *in vitro* and *in vivo* tumor inhibition performance was observed under the electric field. This work demonstrated an alternative concept of electric-enhanced CDT. However, we conceive that there are many safety risks associated with this treatment method and the control of the electric field voltage needs to be strictly controlled, and the stability and side effects of this treatment need to be strictly evaluated.

## 5. Discussion of CDT agents for other biomedical applications

**Fig. 17:**
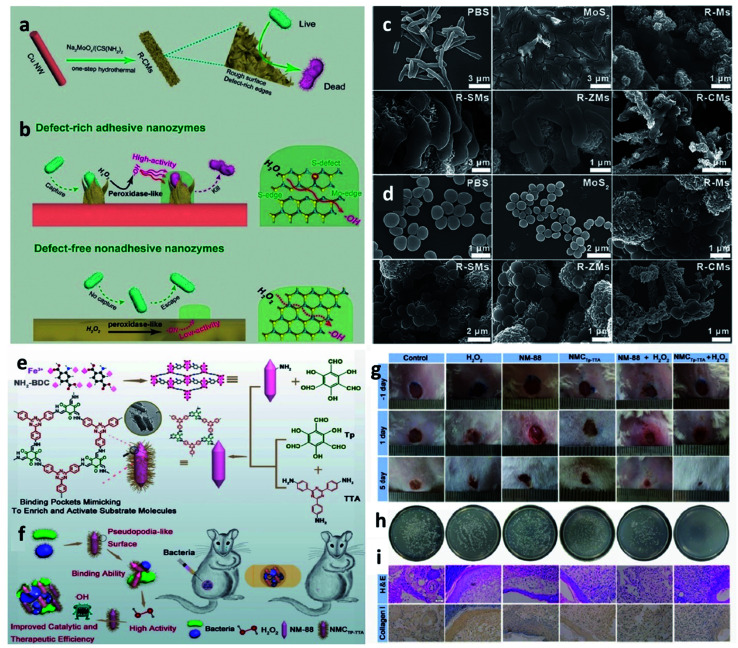
(a and b) Schematic synthetic process of adhesive and defect-rich nanozymes for improved bacterial capture and elimination. (c and d) SEM images of *E. coli* and *S. aureus* after different treatments. Reproduced with permission from ref. 79. Copyright 2019, Wiley-VCH. (e and f) Schematic synthetic process of NMC_Tp-TTA_ (a nature-inspired MOF@COF nanozyme) hybrid nanozyme for bacterial inhibition. (g) Photographs of the infected wounds treated with different groups. (h) Photographs of the bacterial colonies separated from the wounds treated with different groups. (i) H&E and collagen I staining of skin tissues from mice. Scar bar: 50 μm. Reproduced with permission from ref. 80. Copyright 2020, Wiley-VCH.

To broaden the application area for CDT agents, a brief discussion of CDT agents for other biomedical applications is added for researchers in the other areas, such as antibacterial therapy is introduced for reference. In this section, we will only briefly illustrate how CDT agents can be used in other ROS-mediated therapies, using antimicrobial therapy as an example. For instance, in 2019, Qu *et al.* reported a novel nanozyme with a defect-rich active center and rough surfaces for chemodynamic antibacterial therapy (Fig. 17a–d).^79^ Compared with the pristine nanozyme, the as-prepared nanozymes possess high adsorption energy of H_2_O_2_ and desorption energy of OH*, as well as higher Fenton catalytic activity. Both *in vitro* and *in vivo* experimental results reveal that the as-obtained nanozymes have a broad-spectrum antibacterial ability. This work proves that nanozymes that could effectively activate Fenton reactions could also be used as alternative antibiotics for chemodynamic antibacterial therapy. Moreover, in 2020, they again provided a MOF@COF nanozyme with high-efficiency peroxidase activity for antibacterial therapy inspired by nature (Fig. 17e–i).^80^ Apart from research for applying CDT in the field of anti-bacteria, other therapies based on reactive oxygen species could also consider CDT agents. We conceive that CDT agents would find more adequate biomedical applications with a further understanding of the working mechanism of Fenton chemistry.

## 6. Conclusions and perspectives

We first systematically introduce the working mechanisms of Fenton chemistry associated with cancer chemodynamic therapy, including the characteristics of Fenton nanomaterials and unique features of the tumor microenvironment. Then, we pay our attention to proposing different strategies that aim to optimize the efficiency of Fenton/Fenton-like reaction activities in TME, mainly including the selection and designing of suitable Fenton agents, *in situ* increasing the H_2_O_2_ level in TME, *in situ* decreasing the content of GSH, as well as introducing physical energy fields. We firmly believe that enhanced cancer therapy *via* CDT agents has great potential in cancer treatments. More importantly, against its current infancy of CDT, further refinement of the CDT agents mentioned above is strongly needed and will make that CDT nanoplatform more applicable to biomedical areas. In addition, there are still some significant issues that must be considered carefully to promote the development of CDT in clinic transformation. Current challenges that CDT faces and our representative perspectives are listed as follows.

(1) In-depth understanding and explorations of Fenton and anticancer pathways of CDT at a genetic and molecular level are urgently needed to guide the design of more efficient CDT agents to achieve the optimized CDT effect. (2) The specificity or biocompatibility of CDT must be improved to reduce the side effect. (3) The evaluation of CDT treatment efficiency must be broadened to other animal models even humans, not merely for mice models. (4) CDT agents should be easily modified for different kinds of cancers. (5) The off-targeting rate of CDT agents should be decreased. We can't only rely on passive targeting (EPR effect), active targeting strategies should be considered in the future. (6) The long blood circulation time of Fenton agents is also an important factor that greatly influences the treatment efficiency. (7) To achieve real-time monitor and assessment of CDT effect, advanced imaging methods should be introduced. (8) The current structure-dependent CDT enhancement strategies like disordered atom arrangement and chelating strategy could be also developed. (9) The production yield of current CDT agents in the human body must be increased for practical application. (10) Artificial intelligence technologies, as well as big data technologies, can provide services and useful guidance for the screening of CDT therapeutic agents and the evaluation of post-treatment outcomes. (11) Computer science could be employed to help us to design CDT agents from the angle of theory. (12) Apart from ˙OH, other radical forms, such as sulfur-free radicals, chlorine-free radicals, and so on, should be considered into Fenton chemistry, further developing chemodynamic cancer therapy.

## Data availability

There are no experimental or computational data in this article.

## Author contributions

Changyu Cao: writing-original draft preparation. Xiaorui Wang & Nan Yang: visualization, review & editing. Xuejiao Song: review & editing, funding acquisition. Xiaochen Dong: supervision, review and editing, funding acquisition.

## Conflicts of interest

The authors declare no competing financial interest.

## Supplementary Material
